# From manual counting to YOLO: Using computer vision to automate large-scale fecundity assays in *C. elegans*

**DOI:** 10.1371/journal.pone.0354821

**Published:** 2026-07-30

**Authors:** Linyao Peng, Hongyi Shui, Anne Nicole Janisch, Sarah Dede Hesse, Victoria Kathleen Feist, Amanda Kyle Gibson

**Affiliations:** 1 Department of Biology, University of Virginia, Charlottesville, Virginia, United States of America; 2 Department of Integrated Marketing Communications, Northwestern University, Evaston, Illinois, United States of America; Centre National de la Recherche Scientifique & University of Nice Sophia-Antipolis, FRANCE

## Abstract

Fecundity measurements play a crucial role in life history research, providing insights into reproductive fitness, population dynamics, and environmental responses. In the model nematode *Caenorhabditis elegans*, fecundity assays are widely used to study development, aging, and genetic or environmental influences on reproduction. *C. elegans* hermaphrodites have large numbers of offspring (>100), so manual counting of viable offspring is time-consuming and susceptible to human error. Automated counting methods have the potential to enhance throughput, accuracy, and precision in data collection. We applied computer vision to 9972 images of broods from individual *C. elegans* hermaphrodites from several strains under multiple treatments to capture variation in fecundity. We trained models using You Only Look Once (YOLO) versions v8 to v11 (large and extra-large variants) to detect and count viable offspring, then compared the model results to estimates from manual counting. The best-performing model, YOLO v11-L, detected offspring with high accuracy after fine-tuning, achieving 92.6% recall and 94.9% precision. Manual counts differed from verified ground-truth counts by an average of 2.16 offspring per image, compared to 0.9 for the trained computer vision model. In addition, we detected significant effects of counter identity, experimental block, and their interaction on manual counts. Computer vision counts were not affected by these biases and outperformed manual counting in both speed, consistency, and accuracy. We demonstrate that computer vision can be a powerful tool for fecundity assays in *C. elegans* and provide a pipeline for applying this approach to new image sets. More broadly, applying computer vision to digital collections can advance ecological and evolutionary research by accelerating the study of fitness and life history. In practice, this reduces months of manual counting to about 2 hours on a consumer GPU, lowering barriers to large-scale fecundity assays.

## Introduction

Fecundity assays are a fundamental approach in ecology and evolution [1–5]. They provide measurements of reproductive output, population dynamics, and fitness [6–9]. They are routinely used to measure the sensitivity of reproductive success to environmental stressors, including variation in temperature, pollution, parasitism, and exposure to chemicals [10–13].

Fecundity assays are especially popular in research with the model nematode *Caenorhabditis elegans*. An individual’s total reproductive output can be tracked over the course of ~5 days and used to estimate key life history parameters [14–17]. These studies tackle important questions on the impact of standing genetic variation, mutations, diet and environmental stressors on reproductive success [18,19]. *C. elegans* also serves as a model for investigating other life history traits that are tightly coupled to reproduction, such as developmental rate and lifespan [20–22].

Traditionally, fecundity assays in *C. elegans* are conducted manually using dissecting microscopes to count the number of viable offspring deposited by an individual hermaphrodite. *C. elegans* is highly fecund, producing 100–300 offspring quickly, within a few days [23,24]. Thus, these assays provide rich data, but they are also labor-intensive and prone to human error [25–27]. Depending on offspring density, it can take approximately 1–20 minutes to manually count and validate the offspring produced in one day by a single hermaphrodite. Across all experimental replicates, manual counting can thus amount to hundreds of labor hours. These features limit the use of fecundity assays at larger scales, especially in genome-wide association studies and multifactorial tests of environmental stressors. Large-scale fecundity assays have instead been carried out using high-throughput instruments such as the COPAS BIOSORT large particle sorter [28] or WormScan [29]. Although these approaches enable powerful large-scale analyses, such platforms impose cost and accessibility barriers for many laboratories.

Computer vision may provide a cost-effective solution to *C. elegans* fecundity assays. Computer vision has already been used to quantify reproduction and morphology of various nematode species. Deep learning frameworks that utilized convolutional neural networks (CNNs) accurately identified and counted soybean cyst nematode (*Heterodera glycines*) eggs [30] and phenotyped beet cyst nematodes (*Heterodera schachtii*) [31]. More recent efforts have applied YOLO (You Only Look Once)-based models to detect and classify eggs and juveniles of root knot nematode (*Meloidogyne*) species [32] and to develop tools for estimating population sizes of plant-parasitic nematodes [33]. These advancements underscore the potential for computer vision technologies to revolutionize nematode quantification, providing efficient and reliable alternatives to manual counting.

Computer vision models have been increasingly applied to automate counting in *C. elegans*. These models use a range of deep learning approaches, including convolutional neural networks [34,35], YOLO versions [36–38], Mask R-CNN [36,39], and vision transformers [40]. They show high accuracy in detecting, classifying, and tracking individuals across developmental stages and during behavioral assays. The success of these prior models lays the groundwork for the application of computer vision to fecundity assays, via images of broods. Fecundity assays present particular challenges for existing computer vision models, especially when assays are conducted at scale using budget-friendly equipment. Images of broods may not be consistently high quality, offspring are rarely the same size, and they often overlap one another. Offspring are also typically imaged as early as possible, when they are very small, to ensure they are counted before dying, leaving the plate, or reproducing. Thus, we sought to develop a method for robust and accurate nematode counting that is compatible with variable image quality, flexible experimental set-ups, and mixed stage broods, including early life stages.

To accomplish this goal, we used YOLO versions to train models to count offspring in a large collection of images of *C. elegans* broods. We found that the computer vision models provided fast and reliable estimates of fecundity with less bias than manual counting. Our method can easily be modified for use by other nematode researchers.

## Materials and methods

**Fig 1:**
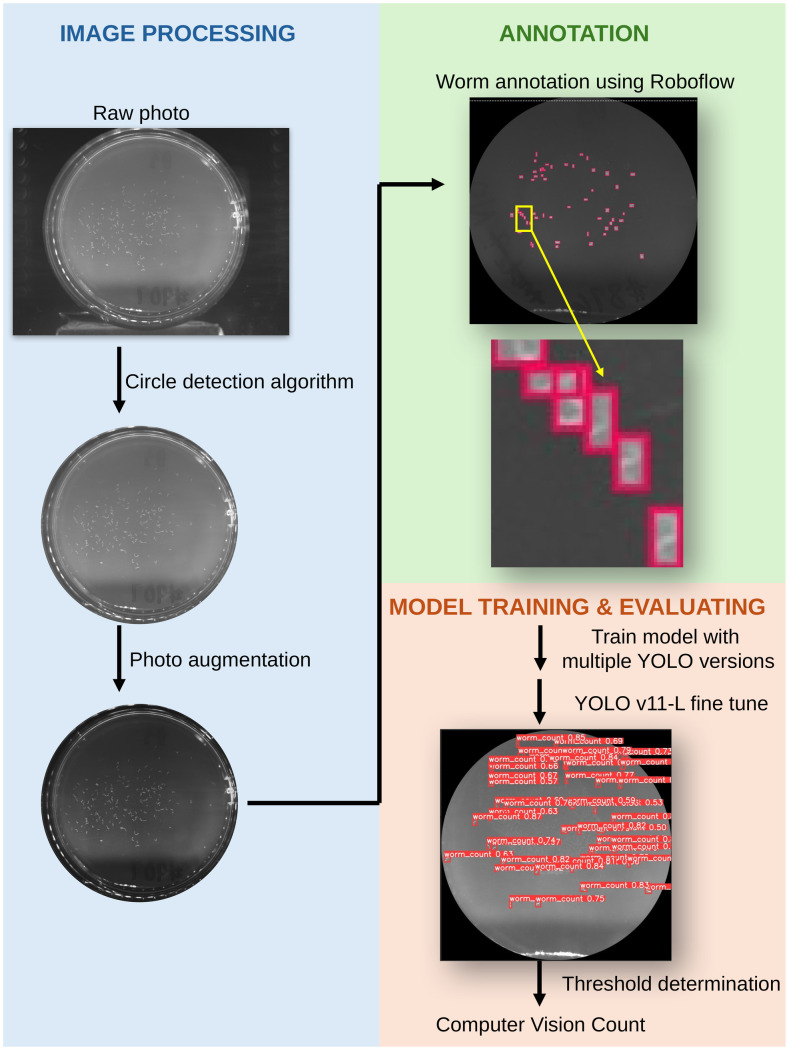
Overview of the complete workflow used for image preprocessing, annotation, model training, and prediction.

This section describes the complete workflow for training and evaluating computer vision models for automatic offspring counting in *C. elegans* fecundity assays. We describe our low-cost imaging setup to generate images, and our approach for designing a custom processing pipeline to isolate regions of interest and standardize image format. We then detail the protocol for annotating images, partitioning the dataset, training models using YOLO model versions, and fine-tuning the top-performing model. Finally, we describe statistical analyses for assessing model accuracy. A schematic of the full pipeline is shown in Fig 1. High resolution example photos are shown in S1-S4 Figs.

### Image acquisition and dataset preparation

#### Equipment.

We built a low-cost imaging station adapted from Churgin and Fang-Yen [41] and optimized for imaging fecundity plates based on Zhang et al. [17]. Imaging was performed using a DMK 33GP031monochrome GigE camera (The Imaging Source) with a 1/ 2.5” Aptina CMOS MT9P031 sensor (2592 X 1944 resolution). Two 1 mm C/CS extension tubes (LAex5, LAex1) were used to adjust the optical path. S2 Table lists all the components, and S5 Fig shows the imaging station. This simple and affordable set up provided reliable and high-resolution images. This imaging station can be modified to provide higher-quality images, but we used this basic version to evaluate the potential for computer vision to perform with images that are low quality and less standardized.

#### Dataset composition.

High-resolution images of broods were captured under controlled laboratory conditions with standardized lighting, positioning, and imaging parameters to ensure consistency across samples. Images were captured using a camera mounted above the sample at a fixed distance sufficient to image the entire 35 mm plate, with illumination provided from below the plate stage, following Churgin and Fang-Yen (41) and Zhang et al. (17). Each image was assigned a unique randomized identification code to facilitate tracking and subsequent annotation processes. The images were of groups of viable offspring hatched and reared on 35 mm petri dishes containing 4 mL of Nematode Growth Medium Lite (NGM Lite) seeded with 20 µL of *E. coli* OP50. Each petri dish was imaged twice to facilitate identification of offspring. Offspring were produced by individual hermaphrodites during a 24h egg-laying period and were incubated at 20°C for 48h after removal of the parent. Imaged offspring were expected to consist primarily of late larval stages through young adults. The individual hermaphrodites were randomly transferred from populations of different *C. elegans* strains that were either unexposed (control) or exposed to *Nematocida parisii* or *Nematocida ironsii*, each at high and low spore doses. These strain and treatment categories were not analyzed as biological variables in this study; rather, they were included to generate a heterogeneous image set spanning a broad range of offspring abundances and plate appearances. The biological effects of strain and treatment are analyzed separately in a related study [42]. The complete dataset comprised 9972 high-resolution images acquired and stored in TIFF format. All images were hand-counted to generate manual count data for subsequent analyses.

#### Image format and pre-processing.

We implemented a custom Python-based preprocessing pipeline to isolate the region of interest (ROI) containing the specimens through the following sequential steps:

1. **Circular Region Detection:** The Hough Circle Transform algorithm was employed to detect and isolate the circular petri dish in each image, establishing the region for subsequent analysis.
2. **Grayscale Conversion and Smoothing:** Images were converted to grayscale and smoothed using a 3 × 3 Gaussian blur kernel, which enhanced specimen-background contrast while reducing noise that could interfere with detection.
3. **ROI Extraction:** A binary mask was generated to isolate the petri dish region, eliminating background elements that could potentially introduce false positives during model inference.
4. **Normalization and Standardization:** The cropped images were resized to maintain dimensional consistency across all samples and saved in PNG format to preserve image quality while reducing file size.

This pre-processing pipeline ensured that YOLO models focused on specimen-containing regions while eliminating potential confounding background artifacts that could compromise detection accuracy.

### Image annotation protocol

A total of 331 images were randomly selected from the complete dataset and manually annotated to create a “ground-truth” image set for model training. For annotation, we used Roboflow [43] as an annotation platform to label all offspring in each image. These ground-truth labels were manually created bounding boxes that designated ‘true’ nematode. Bounding boxes were carefully delineated around each specimen, with particular attention to overlapping specimens, which were individually labeled to ensure comprehensive annotation of all visible organisms. To ensure consistency, a single annotator applied all labels. After annotation, labeled images were manually reviewed for completeness and consistency before being used as ground truth for model training and evaluation. To maintain full control over the data preparation process, we only used Roboflow for annotation. We performed all preprocessing and augmentation of images independently in Python, as described above.

We exported the annotated dataset in PNG, a YOLO-compatible format, including both the image files and label files with normalized bounding box coordinates (center x, center y, width, height). We divided the ground-truth dataset into three non-overlapping partitions: a training set for model training (231 images, 70%), a validation set for hyperparameter optimization and confidence-threshold selection (50 images, 15%), and a test set for final model evaluation and performance assessment (50 images, 15%). The test partition was held out during both model training and confidence-threshold selection and was used only for the final performance evaluations. To ensure balanced and unbiased subsets, we divided the dataset using randomized stratification, a process that preserves the distribution of specimen counts across subsets.

### Model training and fine-tuning

#### Initial YOLO model training.

We systematically evaluated multiple pre-trained YOLO deep learning architectures for nematode detection and quantification [44]. We selected YOLO frameworks for this purpose because they can perform object localization and classification in a single forward pass of the neural network, making them particularly efficient for detection tasks in high-throughput biological imaging applications. The implementation was based on the Ultralytics framework with PyTorch [45] as the underlying deep learning library. All eight YOLO variants were initialized from Microsoft COCO-pretrained weights distributed by the Ultralytics framework.

We trained eight state-of-the-art large and extra-large YOLO model variants (YOLOv8-L/X, YOLOv9-C/E, YOLOv10-L/X, YOLOv11-L/X) under identical conditions to enable direct performance comparison. These models are part of the YOLO family of single-stage object detectors [44], implemented through the Ultralytics framework [46]. Sapkota et al. [47] provides a comprehensive review of YOLO developments through v11. All models were trained for 50 epochs with the following standardized hyperparameters: input image size of 1344 × 1344 pixels, batch size of 1, and 8 workers for data loading. The standard YOLO loss function was used for model training. This loss function incorporates localization, classification, and objectness components. We used the Ultralytics ‘auto’ optimizer setting, which selected AdamW for our training set size of 231 images. The initial learning rate was 0.01, and it decayed linearly to a final learning rate of 0.0001 over 50 training epochs. Momentum was set to 0.937, and weight decay to 0.0005. We used a 3-epoch linear warmup, with warmup momentum of 0.8 and warmup bias learning rate of 0.1. Training and evaluation of the YOLO models were conducted on a local workstation equipped with an NVIDIA GeForce RTX 2080 GPU (8GB VRAM) and 15.5 GB RAM. The software environment comprised Python 3.10.12, PyTorch 2.3.1, OpenCV, and CUDA 12.1. To enhance computational efficiency and training stability, batch normalization was applied to stabilize gradients, and mixed precision training was enabled to accelerate computation while maintaining numerical precision.

#### Confidence threshold optimization using five-fold cross-validation.

**Fig 2:**
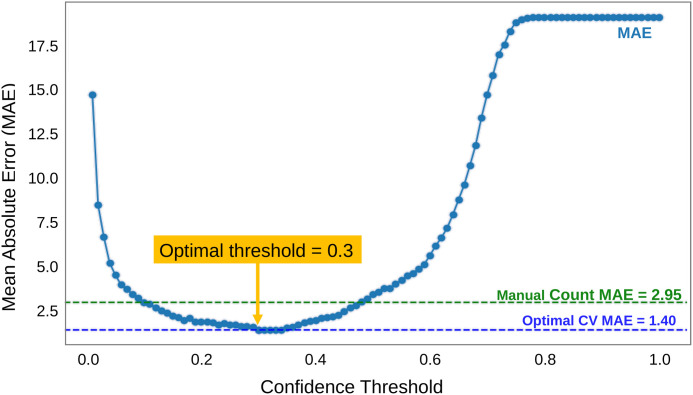
Example chart illustrating the confidence threshold selection process (shown for fold number 5). The green dashed line represents the mean absolute error (MAE) between manual counts and ground truth. The blue dashed line represents the optimal MAE between computer vision (CV) counts and ground truth. The optimal confidence threshold (yellow arrow) corresponds to the x-value at which MAE is minimized (blue dashed line), here at 0.3. The specific optimal threshold and MAE values shown in this figure are for one illustrative fold and should not be interpreted as final Table 1 performance numbers. See Table 1 for final per-model performance on the held-out test set.

A critical parameter in object detection systems is the confidence threshold that determines which predictions are accepted as valid detections. For our purposes, this is the threshold at which an object is counted as a nematode. To systematically identify the optimal confidence threshold for each model, we implemented a rigorous five-fold cross-validation approach. The 50 images in the validation set were divided into five non-overlapping subsets, ensuring each fold contained images with a representative distribution of specimen counts to minimize bias in threshold selection. Confidence thresholds ranging from 0.01 to 1.0 were evaluated at fine-grained 0.01 increments. For each threshold value, the model-generated nematode count (cv_count) was compared against the ground truth count (gt_count). We used mean absolute error (MAE) as the primary optimization metric. MAE is calculated as the average absolute difference between predicted and ground truth counts for an image. For each fold in the five-fold cross-validation approach, we identified the confidence threshold that resulted in the minimum MAE between predicted and ground-truth counts (Fig 2). We then averaged these optimal thresholds across all five folds to determine a final threshold for each model. This approach helped lower the risk of overfitting to any single subset of the data and ensured that the chosen final threshold worked well across a range of image conditions (e.g., varying offspring density per plate).

### Model evaluation and performance metrics

Five-fold confidence threshold optimization allowed us to identify the optimal confidence threshold for each of the eight models trained by the different YOLO variants. We then assessed the detection capabilities of each model at its optimal confidence threshold using a variety of performance metrics to compare computer vision counts to ground annotations. We followed YOLO standard practice in selecting metrics that are relevant to our primary goal of accurate quantification (Redmon et al. 2016). The performance metrics included:

- **Precision (P)**: The proportion of correctly detected nematodes among all detections, calculated as: P = TP/(TP + FP) where TP represents true positives and FP represents false positives. This metric quantifies the model’s ability to avoid false detections.
- **Recall (R)**: The proportion of actual nematodes correctly detected, calculated as: R = TP/(TP + FN) where FN represents false negatives. This metric quantifies the model’s ability to detect all specimens present in the image.
- **F1-score**: The harmonic mean of precision (P) and recall (R), calculated as: F1 = 2×(P × R)/(P + R). This metric summarizes the trade-off between precision and recall, reflecting both the proportion of nematodes correctly detected and the extent to which true nematodes were missed or falsely identified.
- **Mean Absolute Error (MAE)**: The average absolute difference between predicted and ground truth counts for each image. This metric directly quantifies counting accuracy.

We used these metrics to identify the most suitable model for subsequent optimization.

### YOLOv11-L model fine-tuning

Based on comparison of these performance metrics, the model trained with the YOLOv11-L variant was selected for further optimization to enhance its overall detection capabilities. This ‘fine-tuning’ step refers to this hyperparameter optimization of the model already trained in the previous stage.

We selected values for key hyperparameters to fine-tune the YOLO model’s detection performance. These hyperparameters control the training process and were optimized to achieve the best balance between accuracy and computational efficiency. S1 Table presents the hyperparameters and their respective values evaluated during the fine-tuning process, along with the rationale for their selection.

The fine-tuning procedure was conducted on the YOLOv11-L model pre-trained during the initial training phase. To optimize detection performance, we tested multiple experimental configurations, each corresponding to a different combination of hyperparameter values. For each configuration, the model was trained for 50 epochs with consistent settings: input image dimensions of 1344 × 1344 pixels, batch size of 1, and 8 workers for data loading. A five-fold cross-validation approach was employed for confidence threshold optimization throughout the fine-tuning process to ensure robustness of the results and minimize sensitivity to data partitioning.

To quantify the uncertainty of each model’s performance estimate and to support pairwise comparison between models, we computed non-parametric bootstrap 95% confidence intervals for every metric in Table 1. Per-image true positives, false positives, and false negatives were obtained by greedy matching predicted to ground-truth bounding boxes at an intersection-over-union threshold of 0.5. Per-image absolute count error was calculated as the absolute difference between the predicted count and the ground-truth count for that image. We drew 1,000 bootstrap resamples of the 50 held-out test images with replacement, using a fixed random seed so that pairwise comparisons between models operated on identical resamples. The 2.5th and 97.5th percentiles of each metric across the 1000 resamples constituted the reported 95% CI.

**Table 1:** Performance metrics for each YOLO variant and the fine-tuned YOLOv11-L model on the held-out 50-image test set. Values in brackets are 95% bootstrap confidence intervals from 1000 resamples of the test set with replacement.

Model	Threshold	Precision [95% CI]	Recall [95% CI]	F1 [95% CI]	MAE [95% CI]
YOLOv8-L	0.276	0.937 [0.916, 0.956]	0.896 [0.861, 0.929]	0.916 [0.890, 0.939]	0.82 [0.48, 1.20]
YOLOv8-X	0.296	0.928 [0.899, 0.948]	0.890 [0.849, 0.925]	0.908 [0.878, 0.931]	0.98 [0.62, 1.38]
YOLOv9-C	0.292	0.928 [0.905, 0.951]	0.874 [0.826, 0.920]	0.900 [0.866, 0.928]	1.20 [0.72, 1.70]
YOLOv9-E	0.346	0.928 [0.899, 0.953]	0.896 [0.859, 0.929]	0.912 [0.886, 0.935]	1.00 [0.64, 1.36]
YOLOv10-L	0.262	0.898 [0.852, 0.905]	0.881 [0.857, 0.922]	0.889 [0.863, 0.907]	1.08 [0.74, 1.42]
YOLOv10-X	0.186	0.930 [0.849, 0.888]*	0.850 [0.867, 0.932]*	0.888 [0.863, 0.903]	1.16 [0.76, 1.64]
**YOLOv11-L**	**0.308**	**0.952 [0.929, 0.969]**	**0.896 [0.854, 0.929]**	**0.924 [0.896, 0.942]**	**1.00 [0.62, 1.38]**
YOLOv11-X	0.328	0.915 [0.879, 0.950]	0.858 [0.810, 0.898]	0.886 [0.854, 0.911]	1.40 [0.84, 2.00]
**YOLOv11-L-finetuned**	**0.350**	**0.949 [0.913, 0.963]**	**0.926 [0.880, 0.944]**	**0.937 [0.904, 0.948]**	**0.90 [0.58, 1.24]**

*Note: Asterisks indicate models for which the point estimate falls slightly outside the bootstrap CI due to minor differences between the evaluation pipelines used for point estimates and bootstrap resampling.

### Statistical analyses

A strength of our data set is that each computer vision count is paired with a manual count of the same petri dish. This set up allows us to assess the accuracy and consistency of computer vision-based counts versus manual counts. Manual counts were conducted by three trained human counters. Each petri dish was manually counted twice by a single trained counter, and photos were assigned randomly among the three counters. The computer vision counts were sourced from the fine-tuned Yolo v11-L model applied to each image. Each petri dish was imaged twice, so both images were analyzed by the model, and we used the rounded-up average of the two counts as the final value for analysis. Statistical analyses were performed in R (version 4.4.3) (R Core Team).

To assess the agreement between manual counts and computer vision counts, we calculated the correlation between the two sets of values using a simple linear regression. We further compared agreement and evaluated bias using a Bland-Altman analysis. For each image, we plotted the difference between the manual and computer vision counts against the average of the two methods. We then calculated the mean difference (bias) and the 95% limits of agreement, defined as the mean difference ± 1.96 times the standard deviation of the differences. This plot allowed us to visualize both the typical discrepancy between methods and the range within which most count differences fall.

Computer vision counts may be more accurate than manual counts, but perhaps equally important is their potential to be more consistent than manual counts. Consistency may especially be an issue for large assays with many images, such that manual counts must be conducted by multiple people or by a single person over multiple sessions. To assess consistency in our manual counts, we determined if mean bias differed significantly between counters, and whether the bias among counters was inconsistent (i.e., changed across blocks). We performed a two-way ANOVA with bias as the response variable and block, human counter, and their interaction as the predictors.

## Results

We first evaluated the performance of multiple YOLO variants to identify a suitable base model. We selected YOLOv11-L as the optimal variant and fine-tuned hyperparameters to improve performance. Finally, we compared computer vision counts to manual counts, revealing counter-specific and block-specific biases in manual counts.

### Initial YOLO model evaluation

We compared YOLO variants using precision, recall, F1-scores, and mean absolute error (MAE). The performance metrics for all evaluated YOLO variants are presented in Table 1. An example of standard output performance results from the YOLO model is shown in S6 Fig. Among the eight models initially evaluated, YOLOv11-L demonstrated consistently strong performance across metrics, ranking in the top three across all metrics. Notably both precision (0.952) and recall (0.896) were high, resulting in a strong F1-score (0.924) and low MAE (1.00). For our purposes, both over- and under-counting are problematic, so it is critical to achieve a balance between precision, which minimizes false positives, and recall, which minimizes false negatives.

Other models performed well in some metrics, but not all. For example, YOLOv8-L achieved the best MAE (0.82) and high precision (0.937), with recall (0.896) comparable to YOLOv11-L. Similarly, YOLOv9-E showed comparable precision (0.928) and recall (0.896) to YOLOv11-L, with a slightly lower F1-score (0.912) and similar MAE (1.00). We found that the larger variants of YOLO (i.e., the high-capacity “X” models with more layers and parameters) did not consistently outperform their lighter (“L”) counterparts despite their increased capacity. For instance, YOLOv11-X performed worse than YOLOv11-L across all metrics. This result aligns with previous findings: newer or larger YOLO models do not always surpass older versions in real-world tasks (Jiang & Zhong, 2025), and optimizations often yield larger gains than scaling model size alone (Ali, Bhowmik & Nicol,.2023).

### Fine-tuning

After identifying YOLOv11-L as the most promising variant, we conducted systematic hyperparameter optimization to further enhance its detection capabilities. We tested 192 distinct hyperparameter combinations (2 optimizers × 4 IoU thresholds × 4 HSV_V values × 4 scale factors × 4 perspective coefficients × 4 mosaic probabilities) using the same five-fold cross-validation protocol employed during the initial model assessment (S1 Table).

We identified the optimal configuration to be: SGD (stochastic gradient descent) optimizer with IoU = 0.4, HSV_V = 0.5, scale = 0.2, perspective = 0.001, and mosaic = 0.3. This fine-tuned YOLOv11-L model demonstrated substantial improvements (Table 1). Fine-tuning improved recall (+3.0, 0.896 to 0.926), F1 (+1.4, 0.924 to 0.937), and MAE (−0.10, 1.00 to 0.90); precision remained statistically indistinguishable. This reflects an increase in overall counting accuracy. The fine-tuned model operated at a slightly higher optimal confidence threshold (0.350 vs 0.308 for the base model), suggesting that fine-tuning improved the calibration of the model’s confidence scores, enabling a more selective cutoff without loss of recall. In other words, the fine-tuned model more effectively distinguished true nematodes from background elements.

Bootstrap 95% CIs overlap across the four top-performing models (YOLOv8-L, YOLOv9-E, YOLOv11-L, and the fine-tuned YOLOv11-L) on all four metrics, indicating that model-to-model differences among these top performers fall within the sampling variability of a 50-image test set. The fine-tuned YOLOv11-L has the highest recall (0.926), F1 (0.937) and the second lowest MAE (0.90). We therefore present the fine-tuned YOLOv11-L as our preferred pipeline, while noting that statistical superiority over the top-performing initial variants cannot be established at N = 50 images.

### Comparison of computer vision and manual counts

**Fig 3:**
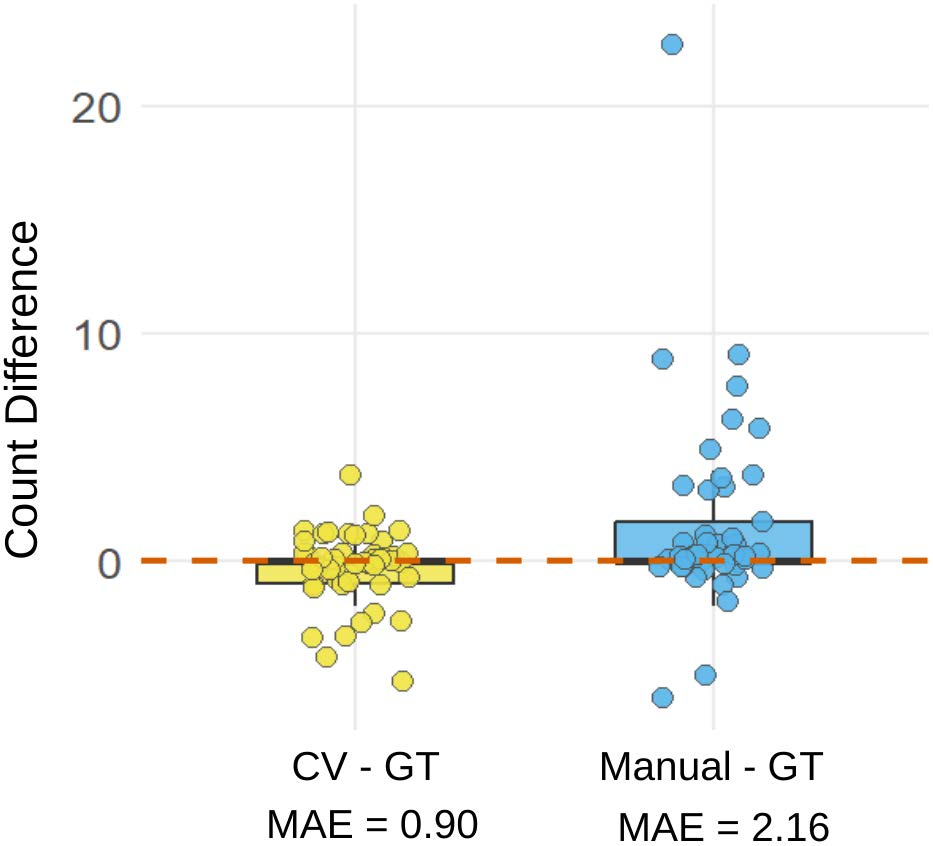
Differences from ground truth counts (GT) for computer vision (CV) and manual counts on the held-out 50-image test set. Computer vision counts reflect the fine-tuned YOLOv11-L model predictions at the held-out-tuned confidence threshold (0.350). Each point represents one image; boxes show interquartile ranges. The dashed line at zero indicates perfect agreement with the ground truth count of an image.

In the held-out 50-image test set, manual counts deviated more from ground truth counts than did computer vision counts. This difference was significant when comparing per-image absolute errors between the two methods (paired two-sided t-test on per-image absolute errors: t(49) =2.44, p = 0.018) (Fig 3). The mean absolute error (MAE) for manual counts was 2.16 nematodes per image, compared to 0.90 nematodes per image for the fine-tuned YOLOv11-L predictions.

**Fig 4:**
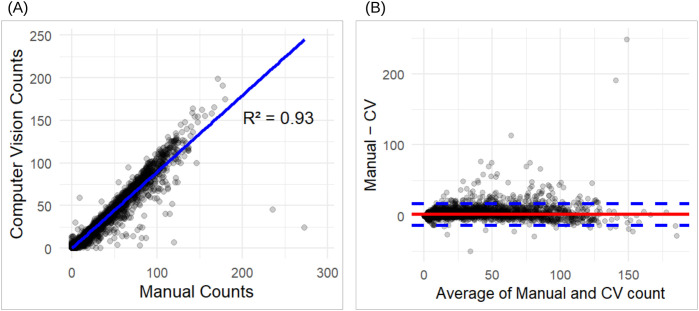
Comparison of manual and computer vision counts. (A) Correlation of computer vision counts and manual counts. Each point represents a single image, and the blue line shows the best-fit linear regression. (B) Bland-Altman plot showing agreement between manual and computer vision counts. Each point represents the difference between a manual count and a computer vision count, plotted against their average. The solid red line indicates the mean difference, and the dashed blue lines represent the 95% limits of agreement.

The fine-tuned YOLOv11-L counts showed a strong linear relationship with manual counts (linear regression: R^2^ = 0.93; Fig 4A), indicating high consistency between the two methods across the range of observed values. Bland-Altman analysis revealed a mean difference of 2.16 nematodes between manual and computer vision counts, suggesting a small positive bias in manual relative to computer vision estimates (Fig 4B). The 95% limits of agreement ranged from −13.51 to 17.84 nematodes, indicating moderate variability in the differences between methods. At higher count values, many points fell outside the agreement bounds, although these cases represented a relatively small proportion of the dataset (1.7%) and not all higher count values lead to higher discrepancies. We examined the total of 84 images with counting discrepancies greater than 21nematodes between manual and computer vision estimates. In 61 of these images, the computer vision counts were more accurate than the manual counts. The remaining 23 images were compromised by either mixed generations of nematodes or poor photo quality.

**Fig 5:**
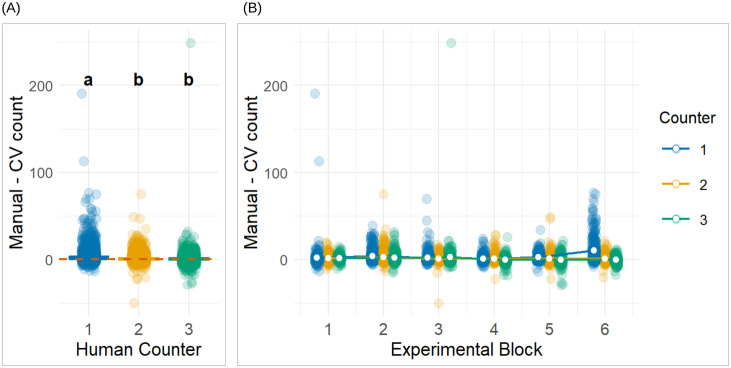
Bias between computer vision and manual counts as a function of counter and block. (A) Counter bias: The difference between manual and computer vision (CV) counts for each human counter, with letters indicating Tukey HSD post hoc comparisons among counters. Each point represents a single image, and the dashed red line at zero denotes perfect agreement between methods. (B) Bias across experimental blocks, showing individual data points and mean trends for each counter.

We observed significant effects of counter identity, experimental block, and their interaction on the bias between manual and computer vision counts (Fig 5). A two-way ANOVA revealed main effects of Counter (F(2, 5013) = 68.32, p < 0.001) and Block (F(5, 5013) =19.47, p < 0.001), as well as a highly significant Counter × Block interaction (F(10, 5013) = 23.66, p < 0.001). Follow-up analyses showed that all three counters produced manual counts that were significantly higher than the corresponding computer vision counts on average: Counter #1 by 3.96 nematodes per image (95% CI [3.47, 4.45], p < 0.001), Counter #2 by 1.50 nematodes per image (95% CI [1.24, 1.76], p < 0.001), and Counter #3 by 1.09 nematodes per image (95% CI [0.73, 1.44], p < 0.001) (Fig 5A). The interaction effect indicates that these patterns of counter bias varied across blocks, which were counted in different weeks over a six-month period (Fig 5B). Together, these results demonstrate that YOLOv11-L-derived counts closely matched to manual counts while offering improved consistency.

## Discussion

In this study, we developed a computer vision pipeline based on the YOLOv11-L deep learning model to count viable offspring in *Caenorhabditis elegans* fecundity assays. Our approach represents a significant innovation by applying object detection to whole-plate images captured using a cheap and user-friendly camera setup. Our model was evaluated against a large, manually curated dataset, enabling a more rigorous comparison to traditional human assessment. We found that our computer vision model performed substantially better than manual counting.

### Advancing life history study in *C. elegans* using computer vision

The most evident advantage of our computer vision model is speed. With the development of machine learning models, computer vision–based object counting has become a powerful approach that dramatically accelerates large-scale assays [48–52]. In our project, manual counting of all 9972 images were distributed across three trained human counters and required approximately 10 minutes per image on average. In total, manual counting took approximately six months. In contrast, computer vision-based counting took a mean of 731ms per image for processing (Hough circle detection, grayscale conversion, 3 × 3 Gaussian blur, region-of-interest masking, and PNG save), and 93ms per image for fine-tuned YOLOv11-L inference on a single consumer-grade NVIDIA RTX 2080 GPU. For the full 9972-image dataset this translates to approximately 2.0 hours of preprocessing and 16 minutes of inference, for a total of approximately 2.3 hours of end-to-end automated processing. This dramatic difference highlights the value of automated approaches for high-throughput quantification. The framework is simple to train and use, and we provide full documentation and guidelines for adapting the approach to other systems in the Data Availability Statement section.

YOLOv11-L also provided more consistent counts than manual counting. In our analysis, computer vision counts avoided the variation that arose among multiple human counters. This consistency is especially valuable for large datasets, which often require multiple counters and may be further complicated if counting practices shift over time. Our computer vision approach thus has the potential to increase throughput and standardize data collection, improving the efficiency and quality of *C. elegans* fecundity measurements. Although the present study was limited to *C. elegans*, the general workflow of image acquisition, manual annotation, and YOLO-based model training could potentially be adapted to other small-bodied organisms or alternative imaging modalities following system-specific retraining and validation.

### Limitations in current *C. elegans* life history research

Early computational efforts with *C. elegans* largely focused on resolving clusters of overlapping nematodes using model-search algorithms [53–55]. With advances in deep learning, particularly convolutional neural networks (CNNs), more recent pipelines have begun incorporating these architectures into nematode detection tasks. Among these, the YOLO family of models has gained prominence for its speed and accuracy in object detection since its introduction in 2015 [56,57]. Thus far, applications of YOLO in *C. elegans* research have primarily focused on behavioral and locomotion analyses [37,58,59], rather than quantification in static images. In contrast, object detection models such as YOLO have been widely used in studies of plant-parasitic nematodes, particularly root-knot and cyst nematodes. These models enable automated classification and quantification of nematodes for use in field diagnostics and agricultural management [60]. This difference in the application of computer vision among nematode models likely reflects research priorities: in agricultural systems, numbers and life history traits of pests directly impact crop yield, driving demand for high-throughput quantification tools.

Broader application of computer vision in life history assays would advance *C. elegans* as a system for addressing key ecological and evolutionary questions. Our dataset pushed the model to perform under realistic and variable biological settings, thereby supporting its application in life history experiments. Our images varied in offspring number, object clustering, and background noise. Offspring counts ranged from dozens to hundreds, with overlapping bodies and subtle morphological variation. Our success given this wide range of image conditions highlights the flexibility of YOLOv11-L to operate in diverse and noisy environments. As such, we hope our pipeline not only bridges a methodological gap but also catalyzes broader adoption of computer vision in *C. elegans* reproductive biology.

### Challenges and future opportunities

While our model showed strong performance, several challenges remain. First, the resolution and quality of images imposed upper limits on detection accuracy. Some photos in our dataset were downsampled due to file size constraints, reducing the model’s ability to resolve tightly clustered or low-contrast individuals. Similar effects of image resolution on object detection performance have been reported in other contexts [61]. Future work could address this by integrating higher-resolution cameras, refining imaging pipelines, or applying super-resolution techniques during preprocessing. These approaches has been shown to enhance small object detection in computer vision tasks [62–64]. However, we purposefully used this low-cost imaging setup to reduce barriers to large-scale fecundity assays, and our model achieved high accuracy nonetheless, demonstrating the practical viability of this approach. The current model was validated for offspring imaged primarily at 48h after egg laying at 20°C, corresponding primarily to late larval stages through young adults. Eggs and earlier larval stages were systematically tested and may require higher-resolution imaging and additional validation. Notably, the largest discrepancies between manual and computer vision counts were concentrated in a relatively small subset of images affected by mixed generations of nematodes or poor photo quality. These cases likely reflect ambiguity in the source images themselves, which can challenge both automated and manual counting. In addition, overlapping or densely clustered worms, debris or other plate artifacts, and variation in lighting or focus may still reduce detection accuracy in some cases. Although the highest-count images in our dataset contained approximately 200 worms, we cannot define a universal upper limit for reliable counting because accuracy depends on abundance, overlap, clustering, image quality, and developmental stage composition. Clustering was not explicitly evaluated in our study, and higher-density or strongly clustered populations should be validated separately. Future work should therefore aim both to improve robustness to these challenging visual conditions and to further standardize the image acquisition process.

Second, improving accessibility was one of our key goals. One distinct advantage of our approach is its ability to run on a standard desktop workstation, avoiding the need for high-end computational resources. This contrasts with many large-scale object detection applications, which typically rely on commercial servers or cloud platforms to handle training and inference at scale [65–67]. By eliminating this barrier, our method is more practical for widespread use in academic laboratories with limited infrastructure. To further enhance accessibility, we envision packaging the model into a user-friendly graphical user interface (GUI), enabling researchers without coding experience to upload images, perform automated analyses, and export fecundity estimates. Such a tool would significantly lower the barrier to application, particularly in life history and evolutionary biology studies using *C. elegans*.

## Conclusion

Together, our results demonstrate the feasibility and value of automated object detection in quantifying reproductive output in *C. elegans*. By combining deep learning with high-resolution life history data, we present a scalable and accurate alternative to manual counting. For a 9972-image dataset, this pipeline reduced quantification time from approximately six months of distributed manual labor to about 2.3 hours on a single consumer-grade GPU, making large-scale life-history experiments feasible without specialized high-throughput phenotyping equipment.

## Supporting information

S1 Table Hyperparameters evaluated during YOLOv11-L fine-tuning. (XLSX)

S2 Table Components of the camera imaging station setup. (XLSX)

S1 Fig Example of a raw image in TIFF format. (TIF)

S2 Fig Example of image in S1 Fig, after circular region detection, grayscale conversion and smoothing, and ROI Extraction. (TIF)

S3 Fig Example of an image annotated with RoboFlow. (TIF)

S4 Fig Example of image in S1 and S2 Figs using the YOLOv11-L-finetuned model with optimal threshold. All predicted nematodes have scores above threshold and are highlighted by bounding boxes.
(TIF)

S5 Fig Imaging station. The design was adapted from Churgin and Fang-Yen (2015).
(TIF)

S6 Fig Example of standard output performance results from the YOLO model (shown for YOLO v11-L). Training and validation performance metrics across epochs are shown, including box loss, classification loss, and distribution focal loss (DFL) (top and bottom left panels), along with precision, recall, mean average precision at IoU ≥ 0.5 (mAP50), and mean average precision across IoU thresholds from 0.5 to 0.95 (mAP50–95) (right panels). Blue lines indicate raw results, and dashed orange lines indicate smoothed trends.
(TIF)
