# Supplementary figures and images for: From manual counting to YOLO: Using computer vision to automate large-scale fecundity assays in C. elegans
Source: PLoS One. 2026 Jul 30;21(7):e0354821. doi: 10.1371/journal.pone.0354821 (PMC13423053; doi:10.1371/journal.pone.0354821)

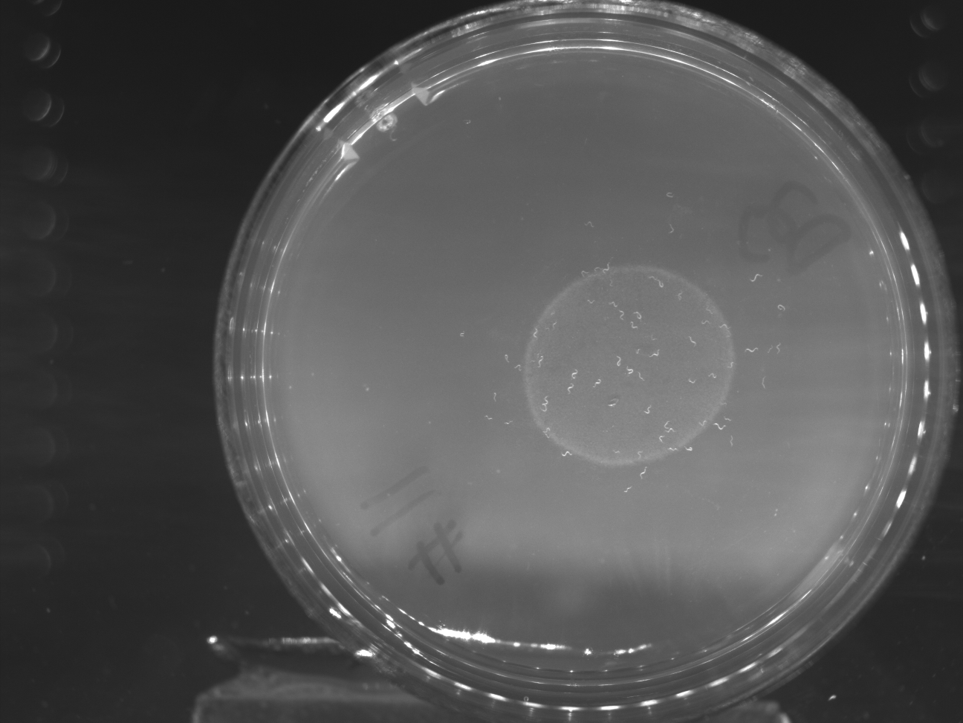

Supplement: S1 Fig — (TIF) [file pone.0354821.s003.tif]

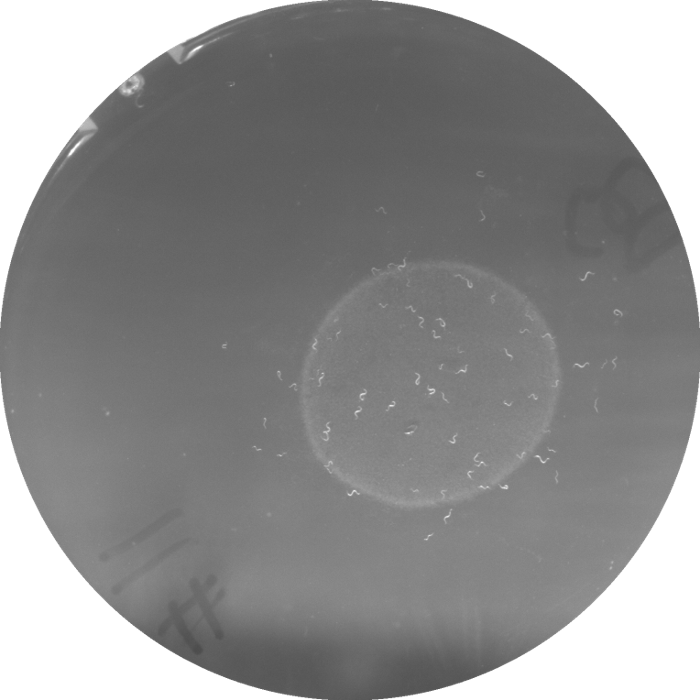

Supplement: S2 Fig — (TIF) [file pone.0354821.s004.tif]

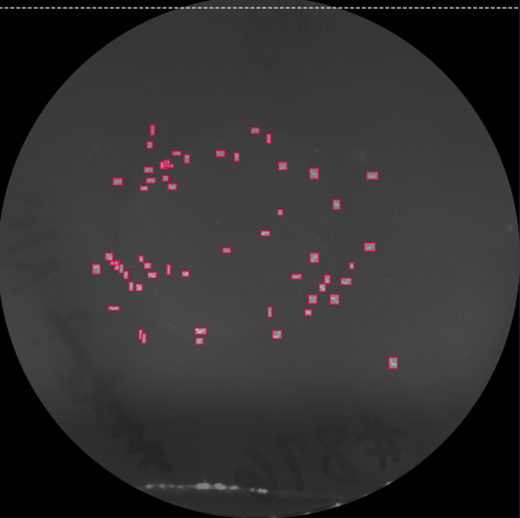

Supplement: S3 Fig — (TIF) [file pone.0354821.s005.tif]

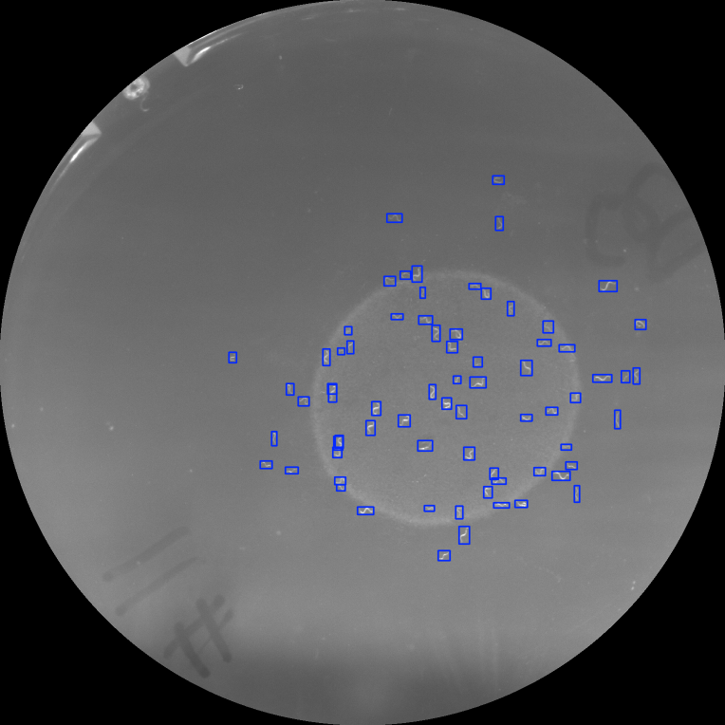

Supplement: S4 Fig — All predicted nematodes have scores above threshold and are highlighted by bounding boxes. (TIF) [file pone.0354821.s006.tif]

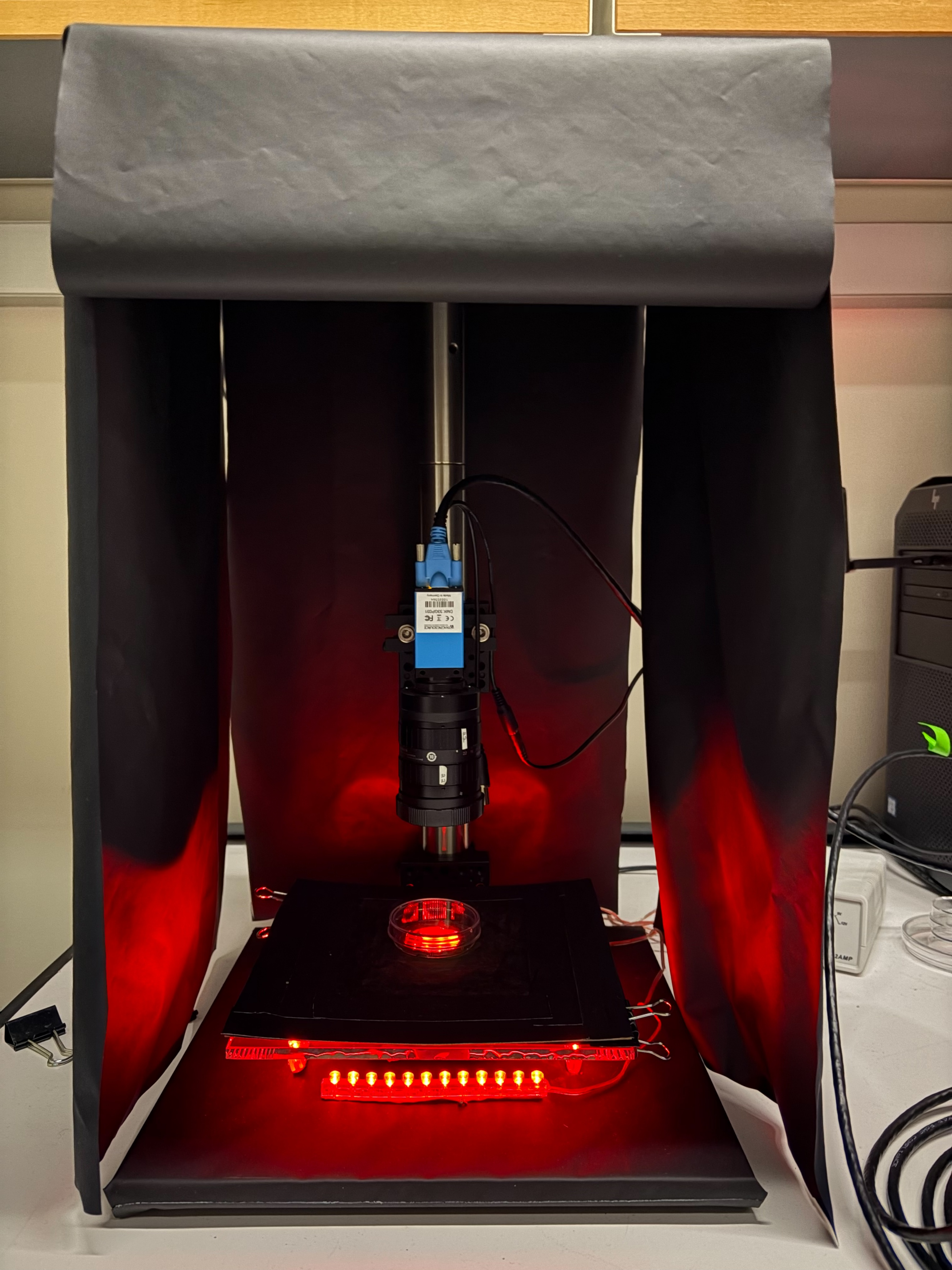

Supplement: S5 Fig — The design was adapted from Churgin and Fang-Yen (2015). (TIF) [file pone.0354821.s007.tif]

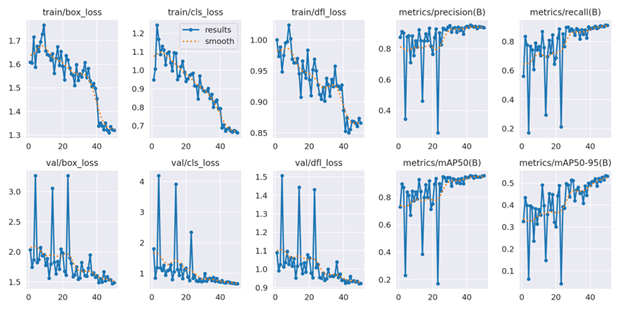

Supplement: S6 Fig — Training and validation performance metrics across epochs are shown, including box loss, classification loss, and distribution focal loss (DFL) (top and bottom left panels), along with precision, recall, mean average precision at IoU ≥ 0.5 (mAP50), and mean average precision across IoU thresholds from 0.5 to 0.95 (mAP50–95) (right panels). Blue lines indicate raw results, and dashed orange lines indicate smoothed trends. (TIF) [file pone.0354821.s008.tif]
