# Supplementary material for: From manual counting to YOLO: Using computer vision to automate large-scale fecundity assays in C. elegans
Source: PLoS One. 2026 Jul 30;21(7):e0354821. doi: 10.1371/journal.pone.0354821 (PMC13423053; doi:10.1371/journal.pone.0354821)
Supplement: Attachment — Submitted filename: Response_to_Reviewers.docx [file pone.0354821.s010.docx]

**Responses to Journal Requirements**

1. Style requirements and file naming

The manuscript and files have been revised to conform to PLOS ONE style and file-naming requirements.

2. Code-sharing requirements

We have reviewed the PLOS ONE code-sharing guidelines and revised our code and related materials accordingly. All author-generated code underlying the findings will be made available without restriction upon publication.

3. Role of Funder statement

We have added the amended Role of Funder statement to the cover letter as requested: “The funders had no role in study design, data collection and analysis, decision to publish, or preparation of the manuscript.”

4. Funding information / Acknowledgments

Funding-related text has been removed from the Acknowledgments section of the manuscript. The amended funding information has been provided in the cover letter so that the online Funding Statement can be updated accordingly.

5. Supporting Information captions

Captions for all Supporting Information files have been added at the end of the manuscript, and in-text citations have been updated to match.

6. Unreadable R file

The Supporting Information file “analysis and figure making scripts.R” has been revised and re-uploaded in an accessible format.

7. Reviewer-suggested citations

No specific additional citations were recommended by the reviewers.

**Response to Reviewer 1**

#1: “The abstract reports performance metrics (92% recall and 94.6% precision), but does not state whether metrics were computed on the same dataset used for training or on a held-out test set. This is crucial for assessing generalizability.”

Response to #1: We thank the reviewer for this important point. Upon re-examination of our original pipeline, we realized that the confidence threshold was selected via 5-fold cross-validation on the combined 100-image validation + test partition, and that the metrics reported in the Abstract and Results were computed on the same 100-image partition. This partition was technically disjoint from the training set, but it was not strictly held out from all phases of model development, which is not ideal. We have accordingly corrected the evaluation protocol: the confidence threshold is now selected by 5-fold cross-validation on the 50-image validation partition only, and all metrics reported in the revised Abstract and Table 1 are computed on the 50-image test partition, which was not used at any stage of training or threshold selection.

We clarified our approach in the Methods [Lines 169-177]. In the Results, we updated the performance metrics reported in Table 1; the updated numbers are very similar to those reported in our original submission (fine-tuned YOLOv11-L: precision 0.946 → 0.949, recall 0.921 → 0.926, MAE 0.95 → 0.90). We have additionally added 95% bootstrap confidence intervals for every metric in Table 1, responding to the reviewer’s separate request for statistical quantification (Point #6).

We also re-did the comparison of manual and computer vision counts using the same 50-image held-out test set. Consistent with our prior findings, this analysis with the test set shows that computer vision counts were significantly more accurate than manual counts (paired two-sided t-test on per-image absolute errors (t(49) = 2.44, p = 0.018)). We calculated a manual mean absolute error (MAE) of 2.16 versus 0.90 for computer vision [Line 354]. Figures 3, 4, and 5, along with the associated Results text, have been updated to reflect these revised analyses.

#2: “The introduction describes limitations of manual fecundity assays but lacks a quantitative estimate of time or labor burden. Providing even approximate labor hours per 1,000 images would contextualize the benefit of automation.”

Response to #2: Thank you for this suggestion. We agree that including a quantitative estimate of manual counting effort improves the framing of the manuscript. We therefore added text to the Introduction stating that, depending on offspring density, manual counting and validation can take approximately 1 - 20 minutes per image, corresponding roughly to hundreds of labor hours per 1000 images [Line 61-63]. We also added a dataset-specific estimate to the Discussion: in this project, manual counting of 9972 images by three trained human counters required approximately 10 minutes per image on average and extended over approximately six months, whereas computer vision-based counting required a mean of 731 ms per image [Line 407-415].

#3: “The dataset description mentions multiple strains and treatments but does not enumerate how many images correspond to each strain/treatment group. This raises concerns about class imbalance and domain shift.”

Response to #3: We agree that the role of host strain and parasite treatment in this study was not sufficiently explained in the original manuscript. In the present work, strain identity and treatment were not prediction targets and were not analyzed as biological factors affecting model performance. Rather, these images were drawn from a broader experimental dataset that includes multiple host strains and parasite treatments, which were analyzed separately for another manuscript (Preprint doi: 10.64898/2026.02.28.708748). Images were balanced across conditions, with offspring counted for ~10 hosts per strain per treatment. For the purposes of the present study, inclusion of these conditions allowed us to assemble a heterogeneous image set spanning a broad range of offspring abundances, including images ranging from zero to several hundred worms. This diversity was useful for evaluating the computer vision pipeline across a wide range of counting scenarios. We have revised the manuscript [Line 134-136] to clarify that the goal of this paper is to present and evaluate the computer vision counting method relative to manual counting, rather than to test biological effects of strain or treatment.

#4: “The manuscript does not specify magnification, camera resolution, lighting conditions, or plate background variability, all of which influence YOLO detection reliability and transferability.”

Response to #4: We now state the imaging more explicitly. The imaging setup did not use microscope optics or variable magnification; instead, images were acquired using a fixed whole-plate configuration in which a camera mounted above the sample captured the full 35 mm plate at a constant camera-to-plate distance. Illumination was provided from below the plate stage, following the imaging approach described by Churgin and Fang-Yen [41] and Zhang et al. [17]. We have clarified these acquisition conditions in the Methods [Line 125-127], in addition to the camera model and resolution already provided (Table S1 and Figure S5), to make the imaging setup and its consistency across samples clearer.

#5: “The training procedure lacks detail: augmentation types, optimizer, learning rate schedule, batch size, and number of training epochs are not stated. Without these, the study is not reproducible.”

Response to #5: We have expanded the Methods section [Line 192-193; 201–205] for the initial training description, with all values taken directly from the saved training run artifacts. The revised Methods now specify that the models were trained for 50 epochs with a batch size of 1 and an input image size 1344 × 1344 pixels. Models were initialized from Microsoft COCO-pretrained weights distributed by the Ultralytics framework. We used Ultralytics ‘auto’ optimizer selection, which for our 231-image training set resolves to AdamW with initial learning rate of 0.01 and linear decay to a final learning rate of 0.0001. Momentum was set to 0.937, and weight decay to 0.0005, with a 3-epoch linear warmup.

#6: “YOLOv8 and YOLOv11 models are compared, but no statistical comparison (e.g., confidence intervals) is provided to determine whether observed improvements are meaningful and not within noise.”

Response to #6: We agree that point estimates alone are insufficient for a between-model comparison. We have computed 95% bootstrap confidence intervals for Precision, Recall, F1, and MAE of every model in Table 1 (1000 resamples of the 50-image held-out test set with replacement, with a fixed random seed so pairwise comparisons use identical resamples). We revised the Methods section to include a new statistical comparison paragraph, inserted after line 277 (end of the existing Statistical Analyses section), where we describe this procedure. The revised Table 1 reports each metric as a point estimate with its 95% CI. The 95% CIs of the top four models, YOLOv8-L, YOLOv9-E, YOLOv11-L, and the fine-tuned YOLOv11-L, overlap on all four metrics, and we inserted a new paragraph reporting this after the end of the Fine-tuning Results subsection [Line 350-358]. We explicitly note that statistical superiority over the top initial variants cannot be established with our 50-image test set, but we present the fine-tuned YOLOv11-L as our preferred pipeline on the basis of its consistent point-estimate ranking across Recall, F1, and MAE. This framing, in which model ranking is backed by explicit uncertainty estimates rather than point estimates alone, substantially improves the paper.

#7, #8, #9, #10: “The manuscript states that verified ground-truth counts exist but does not describe: How were annotations validated? Was inter-annotator agreement measured? This is critical when using biological imagery.”

Response to #7,8,9,10: Thank you for this important comment. We agree that the original manuscript did not clearly distinguish between annotation generation and annotation validation. We have revised the Methods to clarify that the ground-truth image set was annotated by a single annotator using manually drawn bounding boxes around each visible offspring, including overlapping individuals labeled separately. After annotation, the labeled images were manually reviewed for completeness and consistency, with particular attention to densely populated images and overlapping worms, before being used for model training and evaluation. Because all annotations were performed by a single annotator to maintain consistency in labeling criteria, inter-annotator agreement was not measured. We now state this explicitly in the revised manuscript [Line 166-169].

#11: “The manuscript shows counter-identity bias but does not clarify how many counters participated, nor whether counters were randomized or assigned systematically across blocks.”

Response to #11: Thank you for this comment. The original manuscript stated that manual counts were conducted by three trained human counters [Line 283], and we have added further information about how annotators were assigned. We have revised the Methods to clarify that each petri dish was manually counted once by a single counter and that dishes were assigned randomly among counters rather than systematically by experimental block [Line 284-286]. This additional clarification should make the basis of the counter-identity analysis clearer.

#12: “The claim that the YOLO model ‘outperformed manual counting in both speed and consistency’ is asserted but the manuscript does not quantify time saved (e.g., minutes per plate vs. seconds per inference).”

Response to #12: We have added a concrete quantitative comparison to the Discussion [Line 407-415]. The automated pipeline was benchmarked on the actual workstation (NVIDIA RTX 2080, CUDA 12.1, PyTorch 2.1, Ultralytics 8.3.64). Preprocessing, which includes Hough circle detection, grayscale conversion, 3 × 3 Gaussian blur, region-of-interest masking, and PNG save, as described in Methods lines 133–150, takes a mean of 731 ms per image (benchmarked on a random sample of 20 raw TIFFs after warmup). Fine-tuned YOLOv11-L inference takes a mean of 93 ms per image (batch size 1, image size 1344 × 1344; benchmarked on the 50 held-out test images after warmup). For the full 9,972-image dataset this corresponds to approximately 2 hours of preprocessing and 16 minutes of inference, for a total of approximately 2.3 hours of end-to-end automated processing. In comparison, manual counting of the same dataset was distributed across three trained human counters over approximately six months at approximately 10 minutes per image (including counting, examination of data, discussion, recounting).

#13: “The manuscript asserts that the pipeline is generalizable to other organisms, yet no evidence or demonstration is provided beyond C. elegans — this should be presented as a hypothesis, not a conclusion.”

Response to #13: agree that the original wording was too strong given that the present study evaluated the pipeline only using *C. elegans* images. We have revised the manuscript to present broader applicability as a hypothesis rather than as a demonstrated conclusion [Line 424-427]. Our goal here was to show that a YOLO-based object detection workflow can perform well for small-animal life-history assessment in a non-microscopic imaging setting. Because the workflow relies on general steps (image acquisition, manual annotation, and model training) rather than *C. elegans*-specific assumptions, we expect that the same general approach could be adapted to other systems after appropriate retraining and validation. Potential examples include related nematodes and other small-bodied organisms with similar imaging and counting challenges. We therefore revised the manuscript to clarify that any extension beyond *C. elegans*, including to other organisms or imaging modalities, remains a hypothesis for future work rather than a conclusion supported by the present data.

#14, #15: “The limitations paragraph mentions future extensions but does not discuss failure cases, such as: overlapping worms, debris mis-detection, variation in plate lighting.”

Response to #14, #15: Thank you for pointing this out. We have revised the limitations paragraph in Challenges and Future Opportunities section [Line 461-469] to mention likely failure cases, including overlapping worms, debris mis-detection, and variation in plate lighting or image quality. We also added clarification that the largest count discrepancies in our image set were concentrated in a small subset of images with mixed generations of nematodes or poor photo quality. Because these images were also challenging for manual counting, we note that some residual error likely reflects ambiguity in the source material rather than model-specific failure alone.

#16: “I think, if possible, The study would be significantly stronger with external-lab or field validation to demonstrate robustness across imaging and handling conditions.”

Response to #16: We agree that validation in an external laboratory or field setting would further strengthen the study by testing robustness across a broader range of imaging and handling conditions. The present work was designed to establish and evaluate the computer vision pipeline, so external validation was beyond the scope of this study. That said, we view such validation as an important next step. Other groups have expressed interest in using our approach, and our method was recently cited (e.g., Padilha, Matheus, et al. 2025). We are encouraged by this interest and eager to see the approach evaluated in additional systems and imaging environments in future work.

#17: “Consider defining abbreviations (YOLO, CV, precision/recall) at first use in both Abstract and Main Text.”

Response to #17: We have added the expansion of YOLO to its first occurrence in the Abstract [Line 29]. YOLO was already defined at first use in the Main Text [Introduction, line 74]. Precision and recall are defined in plain language at their first use in the Abstract (‘was correct about X% of the offspring it marked (precision)’ and ‘correctly detected Y% of all offspring visible (recall)’) and formally with equations in the Methods [Lines 237–242]. Mean absolute error (MAE) is defined at first use in the Methods [Line 221, line 247-249]. The abbreviation CV is not used in the Abstract or main text body; it appears only within the figure captions for Figures 3 and 5, where ‘computer vision (CV)’ is defined at first use within each caption.

#18: “Methods section would benefit from a workflow diagram summarizing complete pipeline.”

Response to #18: We agree that a workflow diagram improves accessibility of the Methods. Figure 1 in the original submission was intended to serve this purpose and provides a schematic overview of the full workflow, while Figures S1–S4 show representative high-resolution images. We have clarified this in the revised figure legend so that the role of Figure 1 as the complete pipeline summary is more explicit.

#19: “The Discussion repeats descriptive results that are already stated in Results; consider more synthesis.”

Response to 19: We agree that portions of the original Discussion repeated descriptive results that were already presented in the Results section. In the revised manuscript, we reduced repetition of specific performance metrics and instead rewrote this section to focus more on synthesis, including the practical implications of improved consistency relative to manual counting, the value of standardization for large datasets scored across multiple counters and time periods, and the broader methodological significance of the workflow [Line 419-422]. We believe these revisions strengthen the Discussion by emphasizing interpretation rather than restating results.

#20: “The phrase ‘fine-tuning’ is used but the pre-training dataset (e.g., COCO, ImageNet) is not mentioned.”

Response to #20: We have added the pre-training source to the Methods section (initial training, #5 response): all eight YOLO variants were initialized from Microsoft COCO-pretrained weights (80-class object detection) as distributed by the Ultralytics framework. We have also clarified the usage of “fine-tuning” in the manuscript [Line 254-255], because our use of the term differs from its most common ML-literature meaning. In this paper, “fine-tuning” refers to hyperparameter optimization applied to the YOLOv11-L model that had already been trained in a previous stage on our 231-image *C. elegans* training set. The sequence of stages is therefore: (i) COCO pre-training, performed by Ultralytics and inherited via initialization; (ii) initial domain-specific training on our 231 training images, applied to all eight YOLO variants; and (iii) the hyperparameter-optimization step that the manuscript calls “fine-tuning”, applied only to the best-performing YOLOv11-L checkpoint from stage (ii).

#21: “I think, adding a one-sentence statement of practical impact would improve readability.”

Response to #21: We have added a one-sentence statement to the Conclusion on practical impact [Line 484-487]. We note the improvement from approximately six months of distributed human labor to approximately 2.3 hours of end-to-end automated processing for a 9972-image dataset on a consumer-grade NVIDIA RTX 2080 GPU. We emphasize the accessibility advantage for laboratories without specialized high-throughput instruments. A shorter echo of the same point has also been added as the closing sentence of the Abstract. The specific pipeline runtime comes from the benchmark reported in our response to #12 (preprocessing 731 ms/plate, inference 93 ms/plate, end-to-end total ~2.3 hours for 9,972 images).

**Reviewer #2**

#1: The methodology is generally sound; however, some methodological details could be further clarified to improve reproducibility, particularly regarding the experimental design, parameter settings, and data preprocessing steps.

Response to #1: We thank the reviewer for this positive assessment and constructive suggestion. In response, we have substantially expanded the Methods section to improve reproducibility. The revised Methods now report:

- Imaging acquisition: a fixed whole-plate camera configuration with constant camera-to-plate distance and below-plate illumination, in addition to the camera model and resolution already provided in S1 Table and S5 Fig [Lines 125–127].
- Training procedure: 50 epochs, batch size 1, input size 1344 × 1344 pixels, AdamW optimizer (Ultralytics ‘auto’ selection for our 231-image training set), initial learning rate 0.01 with linear decay to 0.0001, momentum 0.937, weight decay 0.0005, and a 3-epoch linear warmup, with all eight variants initialized from Microsoft COCO-pretrained weights distributed by Ultralytics [Lines 192–193, 201–205].
- Annotation protocol: single-annotator manual bounding-box labeling with post-hoc consistency review, with the rationale for not measuring inter-annotator agreement now stated explicitly [Lines 166–169].
- Counter assignment: three trained human counters, with each image counted once by a single counter and dishes randomized among counters rather than assigned by experimental block [Lines 284–286].
- Data preprocessing: the Hough circle detection, grayscale conversion, 3x3 Gaussian blur, ROI masking, and PNG save steps were already described in Methods Lines 133–150; we now additionally report per-step runtime benchmarks (mean 731 ms/image for preprocessing, 93 ms/image for inference) in the Discussion [Lines 407–415].

#2: The results are presented systematically, but the discussion section could be strengthened by providing deeper interpretation of the findings and comparing them more explicitly with recent studies in the literature.

Response to #2: We thank the reviewer for this helpful suggestion. We revised portions of the Discussion to reduce repetition of the Results and to provide a deeper synthesis of the findings. Specifically, we expanded the interpretation of the practical implications of improved consistency relative to manual counting, the value of standardized scoring for large datasets analyzed across multiple counters and time periods, and the broader methodological significance of the workflow [Lines 419–422]. We also added a quantitative comparison between manual and automated counting to better anchor the practical interpretation of the results [Lines 407–415]. We believe these revisions strengthen the interpretation and comparative framing of the Discussion.

#3: Additionally, some figures and tables would benefit from clearer captions and more detailed explanations in the text.

Response to #3: Thank you for this suggestion. We clarified the legend of Figure 1 to make its role as the complete-pipeline workflow summary explicit. Table 1 has been entirely redone: metrics are now reported on the strictly held-out 50-image test set, with 95% bootstrap confidence intervals for Precision, Recall, F1, and MAE for every model. The Results text was extended with a new paragraph noting that the 95% CIs of the top four models (YOLOv8-L, YOLOv9-E, YOLOv11-L, and the fine-tuned YOLOv11-L) overlap on all four metrics, so we present the fine-tuned YOLOv11-L as our preferred pipeline on the basis of its consistent point-estimate ranking rather than a claim of statistical superiority [Lines 350–358]. Figures 3, 4, and 5 were updated to reflect the held-out evaluation, and the associated Results text was revised accordingly. We hope these clarified captions and expanded in-text explanations address the reviewer’s concern.

#4: The manuscript would also benefit from minor language editing to improve clarity and readability.

Response to #4: We have revised the manuscript text in response to the substantive comments above and believe these revisions improve overall clarity and readability.

#5: Expanding the conclusion to better emphasize the practical implications and limitations of the study would further strengthen the manuscript.

Response to #5: We have expanded both the practical implications and the limitations content of the Conclusion. We added a one-sentence statement of practical impact to the Conclusion [Lines 484–487] and a shorter echo to the closing of the Abstract, quantifying the improvement from approximately six months of distributed manual counting to approximately 2.3 hours of end-to-end automated processing for a 9,972-image dataset on a consumer-grade NVIDIA RTX 2080 GPU, and emphasizing the accessibility advantage for laboratories without specialized high-throughput instruments. We also expanded the Challenges and Future Opportunities section [Lines 461–469] to discuss specific failure modes (overlapping worms, debris mis-detection, and variation in plate lighting or image quality) and to note that the largest count discrepancies in our image set were concentrated in images with mixed generations of nematodes or poor photo quality, suggesting that some residual error reflects ambiguity in the source material rather than model-specific failure alone. The cross-organism generalizability statement was softened to a hypothesis for future work [Lines 424–427]. Together, these revisions strengthen the Conclusion’s treatment of both practical implications and limitations.
