# Supplementary material for: From manual counting to YOLO: Using computer vision to automate large-scale fecundity assays in C. elegans
Source: PLoS One. 2026 Jul 30;21(7):e0354821. doi: 10.1371/journal.pone.0354821 (PMC13423053; doi:10.1371/journal.pone.0354821)
Supplement: Attachment — Submitted filename: 2ndresponse_to_reviewers.docx [file pone.0354821.s011.docx]

**Response to Reviewer 3**

#1: “I don't believe the authors ever address limitations on what life stages can be imaged using this method. I assume that they are waiting until a set of eggs laid over 24 hours then develops into adults, because adults are easiest to image, but I wonder if earlier or mixed stages would be quantifiable as well. I'd be interested to see at what stage the reproducibility starts dropping off, even anecdotally, but at the very minimum I think it is important to mention that this was all done in adults (if that is true), so that people understand the limits of what's been tested.”

Response to #1: We thank the reviewer for this helpful suggestion. We agree that the developmental stage of the worms is important to clarify. In our assay, viable offspring were produced by a single adult over a 24-hour egg-laying period. After the parent was removed, plates were incubated at 20℃ for an additional 48 hours before imaging. Because eggs were laid throughout the 24-hour period, the imaged offspring were not perfectly synchronized, but they were sufficiently synchronized such that images did not contain multiple life stages. The imaged offspring consisted primarily of late larval stages and young adults/adults, but some images contained smaller/younger larvae.

We have revised the manuscript [Line 125-128; Line 449-452] to clarify the developmental stage range represented in our dataset. We also now clearly state that the current model was validated for relatively large, post-embryonic worms and was not systematically validated for eggs, early larval stages, or highly mixed-stage populations. We also added a comment that substantially smaller worms would likely require improved imaging resolution, such as a higher-resolution camera, modified imaging setup, or microscope-based imaging, as well as additional training and validation data before the approach could be applied confidently to those stages.

#2: “Is there an upper limit for how many worms can be quantified on a plate? It seems that the images used in this manuscript cap out at about 200 worms, but I wonder if the authors can provide any guidance (again, even just anecdotally) on when counts become unreliable because of overlapping worms -- either due to large population sizes or clustering phenotypes seen in many wild isolates.”

Response to #2: We thank the reviewer for raising this important practical point. We agree that worm density and clustering could affect counting accuracy, especially when individuals overlap extensively. In our dataset, the highest-count images contained approximately 200 worms per plate/image, so we do not claim that the current model has been validated beyond this range or that our dataset can define a universal upper limit for reliable counting. However, However, our inspection of images with the largest discrepancies between manual and computer vision estimates suggests that, within the range tested here, reduced accuracy was driven more by image quality, focus, and mixed-generation images containing very small larvae than by worm abundance alone [Lines 367–371]. We have revised the Discussion to clarify that counting accuracy may depend on abundance, overlap, clustering, image quality, focus, and developmental stage composition, and that higher-density plates or strongly clustered populations should be validated separately using representative images [Lines 458–464].

#3: “There are many values listed on lines 195-198 that have numbers but no units. Where possible, it may be useful to the reader to include units.”

Response to #3: We thank the reviewer for pointing this out. We have reviewed the values in this section and clarified the units where applicable. Specifically, we revised the text to indicate that the learning rate decay occurred over 50 training epochs and that the linear warmup was conducted over 3 epochs. The other values listed in this section, including learning rate, momentum, weight decay, warmup momentum, and warmup bias learning rate, are standard dimensionless model-training parameters, so no additional units were added for those values.
