# Supplementary material for: From manual counting to YOLO: Using computer vision to automate large-scale fecundity assays in C. elegans
Source: PLoS One. 2026 Jul 30;21(7):e0354821. doi: 10.1371/journal.pone.0354821 (PMC13423053; doi:10.1371/journal.pone.0354821)
Supplement: Peer review history

# Peer review: Decision Letter 0

**DOI:** 10.1371/journal.pone.0354821.r001

**Authors:** Ashish Kumar Singh

16 Mar 2026

PONE-D-25-54218

From manual counting to YOLO: Using computer vision to automate large-scale fecundity assays in C. elegans

PLOS One

Dear Dr. Peng,

Thank you for submitting your manuscript to PLOS ONE. After careful consideration, we feel that it has merit but does not fully meet PLOS ONE’s publication criteria as it currently stands. Therefore, we invite you to submit a revised version of the manuscript that addresses the points raised during the review process.

Please submit your revised manuscript by Apr 30 2026 11:59PM. If you will need more time than this to complete your revisions, please reply to this message or contact the journal office at plosone@plos.org. When you're ready to submit your revision, log on to https://www.editorialmanager.com/pone/ and select the 'Submissions Needing Revision' folder to locate your manuscript file.

Please include the following items when submitting your revised manuscript:

- A letter that responds to each point raised by the academic editor and reviewer(s). You should upload this letter as a separate file labeled 'Response to Reviewers'.
- A marked-up copy of your manuscript that highlights changes made to the original version. You should upload this as a separate file labeled 'Revised Manuscript with Track Changes'.
- An unmarked version of your revised paper without tracked changes. You should upload this as a separate file labeled 'Manuscript'.

If you would like to make changes to your financial disclosure, please include your updated statement in your cover letter. Guidelines for resubmitting your figure files are available below the reviewer comments at the end of this letter.

If applicable, we recommend that you deposit your laboratory protocols in protocols.io to enhance the reproducibility of your results. Protocols.io assigns your protocol its own identifier (DOI) so that it can be cited independently in the future. For instructions see: https://journals.plos.org/plosone/s/submission-guidelines#loc-laboratory-protocols. Additionally, PLOS ONE offers an option for publishing peer-reviewed Lab Protocol articles, which describe protocols hosted on protocols.io. Read more information on sharing protocols at https://plos.org/protocols?utm_medium=editorial-email&utm_source=authorletters&utm_campaign=protocols.

We look forward to receiving your revised manuscript.

Kind regards,

Ashish Kumar Singh

Academic Editor

PLOS One

Journal Requirements:

When submitting your revision, we need you to address these additional requirements.

1. Please ensure that your manuscript meets PLOS ONE's style requirements, including those for file naming. The PLOS ONE style templates can be found at

https://journals.plos.org/plosone/s/file?id=wjVg/PLOSOne_formatting_sample_main_body.pdf and

https://journals.plos.org/plosone/s/file?id=ba62/PLOSOne_formatting_sample_title_authors_affiliations.pdf

2. Please note that PLOS One has specific guidelines on code sharing for submissions in which author-generated code underpins the findings in the manuscript. In these cases, we expect all author-generated code to be made available without restrictions upon publication of the work. Please review our guidelines at https://journals.plos.org/plosone/s/materials-and-software-sharing#loc-sharing-code and ensure that your code is shared in a way that follows best practice and facilitates reproducibility and reuse.

3. Thank you for stating the following financial disclosure:

US National Institutes of Health MIRA Award to Amanda K. Gibson (R35GM137975)

Please state what role the funders took in the study. If the funders had no role, please state: "The funders had no role in study design, data collection and analysis, decision to publish, or preparation of the manuscript."

If this statement is not correct you must amend it as needed.

Please include this amended Role of Funder statement in your cover letter; we will change the online submission form on your behalf.

4. Thank you for stating the following in the Acknowledgments Section of your manuscript:

This study was funded by the US National Institutes of Health MIRA Award to Amanda K. Gibson (R35GM137975). The authors thank Erik Andersen (Johns Hopkins University, Department of Biology) for assistance with the camera setup, and all members of the Gibson Lab for their helpful feedback. We are also grateful to Keric Lamb (University of Virginia, Department of Biology) for recommending the statistical analysis used to compare the two counting methods.

We note that you have provided funding information that is not currently declared in your Funding Statement. However, funding information should not appear in the Acknowledgments section or other areas of your manuscript. We will only publish funding information present in the Funding Statement section of the online submission form.

Please remove any funding-related text from the manuscript and let us know how you would like to update your Funding Statement. Currently, your Funding Statement reads as follows:

US National Institutes of Health MIRA Award to Amanda K. Gibson (R35GM137975)

Please include your amended statements within your cover letter; we will change the online submission form on your behalf.

5. Please include captions for your Supporting Information files at the end of your manuscript, and update any in-text citations to match accordingly. Please see our Supporting Information guidelines for more information: http://journals.plos.org/plosone/s/supporting-information.

6. We are unable to open your Supporting Information file [analysis and figure making scripts.R]. Please kindly revise as necessary and re-upload.

7. If the reviewer comments include a recommendation to cite specific previously published works, please review and evaluate these publications to determine whether they are relevant and should be cited. There is no requirement to cite these works unless the editor has indicated otherwise.

Additional Editor Comments:

Dear Authors,

Your manuscript titled “From manual counting to YOLO: Using computer vision to automate large-scale fecundity assays in C. elegans” presents an interesting and potentially impactful application of computer vision for automating fecundity measurements. The effort to reduce manual counting using deep learning approaches is commendable and aligns well with current trends in high-throughput biological data analysis.

The reviewers acknowledge the relevance of your work; however, some issues need to be addressed before the manuscript can be considered for publication. Please carefully revise the manuscript according to the reviewers’ comments.

[Note: HTML markup is below. Please do not edit.]

Reviewers' comments:

Reviewer's Responses to Questions

**Comments to the Author**

1. Is the manuscript technically sound, and do the data support the conclusions?

The manuscript must describe a technically sound piece of scientific research with data that supports the conclusions. Experiments must have been conducted rigorously, with appropriate controls, replication, and sample sizes. The conclusions must be drawn appropriately based on the data presented.

Reviewer #1: Yes

Reviewer #2: Yes

**********

2. Has the statistical analysis been performed appropriately and rigorously?

Reviewer #1: Yes

Reviewer #2: Yes

**********

3. Have the authors made all data underlying the findings in their manuscript fully available?

The PLOS Data policy requires authors to make all data underlying the findings described in their manuscript fully available without restriction, with rare exception (please refer to the Data Availability Statement in the manuscript PDF file). The data should be provided as part of the manuscript or its supporting information, or deposited to a public repository. For example, in addition to summary statistics, the data points behind means, medians and variance measures should be available. If there are restrictions on publicly sharing data—e.g. participant privacy or use of data from a third party—those must be specified.

Reviewer #1: Yes

Reviewer #2: Yes

**********

4. Is the manuscript presented in an intelligible fashion and written in standard English?

PLOS ONE does not copyedit accepted manuscripts, so the language in submitted articles must be clear, correct, and unambiguous. Any typographical or grammatical errors should be corrected at revision, so please note any specific errors here.

Reviewer #1: Yes

Reviewer #2: Yes

**********

5. Review Comments to the Author

Please use the space provided to explain your answers to the questions above. You may also include additional comments for the author, including concerns about dual publication, research ethics, or publication ethics. (Please upload your review as an attachment if it exceeds 20,000 characters)

Reviewer #1: This manuscript presents an automated computer vision workflow using YOLO-based object detection models to quantify C. elegans fecundity from plate images. The authors compare multiple YOLO model variants, evaluate performance against manual counts, and demonstrate that deep learning can reduce labor time and increase consistency in large-scale offspring counting. The study addresses a relevant bottleneck in high-throughput phenotyping and has clear potential impact for experimental genetics and reproductive biology. However, while the research question is valuable and the results are promising, there are several significant issues that need to be addressed before the manuscript can be considered for publication. Below are my comments:

1. The abstract reports performance metrics (92% recall and 94.6% precision), but does not state whether metrics were computed on the same dataset used for training or on a held-out test set. This is crucial for assessing generalizability.

2. The introduction describes limitations of manual fecundity assays but lacks a quantitative estimate of time or labor burden. Providing even approximate labor hours per 1,000 images would contextualize the benefit of automation.

3. The dataset description mentions multiple strains and treatments but does not enumerate how many images correspond to each strain/treatment group. This raises concerns about class imbalance and domain shift.

4. The manuscript does not specify magnification, camera resolution, lighting conditions, or plate background variability, all of which influence YOLO detection reliability and transferability.

5. The training procedure lacks detail: augmentation types, optimizer, learning rate schedule, batch size, and number of training epochs are not stated. Without these, the study is not reproducible.

6. YOLOv8 and YOLOv11 models are compared, but no statistical comparison (e.g., confidence intervals) is provided to determine whether observed improvements are meaningful and not within noise.

7. The manuscript states that verified ground-truth counts exist but does not describe:

8. How were annotations validated?

9. Was inter-annotator agreement measured?

10. This is critical when using biological imagery.

11. The manuscript shows counter-identity bias but does not clarify how many counters participated, nor whether counters were randomized or assigned systematically across blocks.

12. The claim that the YOLO model “outperformed manual counting in both speed and consistency” is asserted but the manuscript does not quantify time saved (e.g., minutes per plate vs. seconds per inference).

13. The manuscript asserts that the pipeline is generalizable to other organisms, yet no evidence or demonstration is provided beyond C. elegans — this should be presented as a hypothesis, not a conclusion. Or Authors should justify this more strongly.

14. The limitations paragraph mentions future extensions but does not discuss failure cases, such as:

15. overlapping worms, debris mis-detection, variation in plate lighting.

16. I think, if possible, The study would be significantly stronger with external-lab or field validation to demonstrate robustness across imaging and handling conditions.

17. Consider defining abbreviations (YOLO, CV, precision/recall) at first use in both Abstract and Main Text.

18. Methods section would benefit from a workflow diagram summarizing complete pipeline.

19. The Discussion repeats descriptive results that are already stated in Results; consider more synthesis.

20. The phrase ‘fine-tuning’ is used but the pre-training dataset (e.g., COCO, ImageNet) is not mentioned.

21. I think, adding a one-sentence statement of practical impact would improve readability.

Reviewer #2: The manuscript addresses an important research topic and presents a relevant contribution to the field. The overall structure of the paper is logical, and the objectives are clearly stated. The introduction provides appropriate background information and highlights the significance of the study. The methodology is generally sound; however, some methodological details could be further clarified to improve reproducibility, particularly regarding the experimental design, parameter settings, and data preprocessing steps. The results are presented systematically, but the discussion section could be strengthened by providing deeper interpretation of the findings and comparing them more explicitly with recent studies in the literature. Additionally, some figures and tables would benefit from clearer captions and more detailed explanations in the text. The manuscript would also benefit from minor language editing to improve clarity and readability. Expanding the conclusion to better emphasize the practical implications and limitations of the study would further strengthen the manuscript. Overall, the study is promising, and the manuscript could make a valuable contribution to the field.

**********

6. PLOS authors have the option to publish the peer review history of their article (what does this mean?). If published, this will include your full peer review and any attached files.

If you choose “no”, your identity will remain anonymous but your review may still be made public.

**Do you want your identity to be public for this peer review?**  For information about this choice, including consent withdrawal, please see our Privacy Policy.

Reviewer #1: No

Reviewer #2: No

**********

[NOTE: If reviewer comments were submitted as an attachment file, they will be attached to this email and accessible via the submission site. Please log into your account, locate the manuscript record, and check for the action link "View Attachments". If this link does not appear, there are no attachment files.]

To ensure your figures meet our technical requirements, please review our figure guidelines: https://journals.plos.org/plosone/s/figures

You may also use PLOS’s free figure tool, NAAS, to help you prepare publication quality figures: https://journals.plos.org/plosone/s/figures#loc-tools-for-figure-preparation.

NAAS will assess whether your figures meet our technical requirements by comparing each figure against our figure specifications.

# Author comment: Author response to Decision Letter 1

**DOI:** 10.1371/journal.pone.0354821.r002

30 Apr 2026

Responses to Journal Requirements

1. Style requirements and file naming

The manuscript and files have been revised to conform to PLOS ONE style and file-naming requirements.

2. Code-sharing requirements

We have reviewed the PLOS ONE code-sharing guidelines and revised our code and related materials accordingly. All author-generated code underlying the findings will be made available without restriction upon publication.

3. Role of Funder statement

We have added the amended Role of Funder statement to the cover letter as requested: “The funders had no role in study design, data collection and analysis, decision to publish, or preparation of the manuscript.”

4. Funding information / Acknowledgments

Funding-related text has been removed from the Acknowledgments section of the manuscript. The amended funding information has been provided in the cover letter so that the online Funding Statement can be updated accordingly.

5. Supporting Information captions

Captions for all Supporting Information files have been added at the end of the manuscript, and in-text citations have been updated to match.

6. Unreadable R file

The Supporting Information file “analysis and figure making scripts.R” has been revised and re-uploaded in an accessible format.

7. Reviewer-suggested citations

No specific additional citations were recommended by the reviewers.

Response to Reviewer 1

#1: “The abstract reports performance metrics (92% recall and 94.6% precision), but does not state whether metrics were computed on the same dataset used for training or on a held-out test set. This is crucial for assessing generalizability.”

Response to #1: We thank the reviewer for this important point. Upon re-examination of our original pipeline, we realized that the confidence threshold was selected via 5-fold cross-validation on the combined 100-image validation + test partition, and that the metrics reported in the Abstract and Results were computed on the same 100-image partition. This partition was technically disjoint from the training set, but it was not strictly held out from all phases of model development, which is not ideal. We have accordingly corrected the evaluation protocol: the confidence threshold is now selected by 5-fold cross-validation on the 50-image validation partition only, and all metrics reported in the revised Abstract and Table 1 are computed on the 50-image test partition, which was not used at any stage of training or threshold selection.

We clarified our approach in the Methods [Lines 169-177]. In the Results, we updated the performance metrics reported in Table 1; the updated numbers are very similar to those reported in our original submission (fine-tuned YOLOv11-L: precision 0.946 → 0.949, recall 0.921 → 0.926, MAE 0.95 → 0.90). We have additionally added 95% bootstrap confidence intervals for every metric in Table 1, responding to the reviewer’s separate request for statistical quantification (Point #6).

We also re-did the comparison of manual and computer vision counts using the same 50-image held-out test set. Consistent with our prior findings, this analysis with the test set shows that computer vision counts were significantly more accurate than manual counts (paired two-sided t-test on per-image absolute errors (t(49) = 2.44, p = 0.018)). We calculated a manual mean absolute error (MAE) of 2.16 versus 0.90 for computer vision [Line 354]. Figures 3, 4, and 5, along with the associated Results text, have been updated to reflect these revised analyses.

#2: “The introduction describes limitations of manual fecundity assays but lacks a quantitative estimate of time or labor burden. Providing even approximate labor hours per 1,000 images would contextualize the benefit of automation.”

Response to #2: Thank you for this suggestion. We agree that including a quantitative estimate of manual counting effort improves the framing of the manuscript. We therefore added text to the Introduction stating that, depending on offspring density, manual counting and validation can take approximately 1 - 20 minutes per image, corresponding roughly to hundreds of labor hours per 1000 images [Line 61-63]. We also added a dataset-specific estimate to the Discussion: in this project, manual counting of 9972 images by three trained human counters required approximately 10 minutes per image on average and extended over approximately six months, whereas computer vision-based counting required a mean of 731 ms per image [Line 407-415].

#3: “The dataset description mentions multiple strains and treatments but does not enumerate how many images correspond to each strain/treatment group. This raises concerns about class imbalance and domain shift.”

Response to #3: We agree that the role of host strain and parasite treatment in this study was not sufficiently explained in the original manuscript. In the present work, strain identity and treatment were not prediction targets and were not analyzed as biological factors affecting model performance. Rather, these images were drawn from a broader experimental dataset that includes multiple host strains and parasite treatments, which were analyzed separately for another manuscript (Preprint doi: 10.64898/2026.02.28.708748). Images were balanced across conditions, with offspring counted for ~10 hosts per strain per treatment. For the purposes of the present study, inclusion of these conditions allowed us to assemble a heterogeneous image set spanning a broad range of offspring abundances, including images ranging from zero to several hundred worms. This diversity was useful for evaluating the computer vision pipeline across a wide range of counting scenarios. We have revised the manuscript [Line 134-136] to clarify that the goal of this paper is to present and evaluate the computer vision counting method relative to manual counting, rather than to test biological effects of strain or treatment.

#4: “The manuscript does not specify magnification, camera resolution, lighting conditions, or plate background variability, all of which influence YOLO detection reliability and transferability.”

Response to #4: We now state the imaging more explicitly. The imaging setup did not use microscope optics or variable magnification; instead, images were acquired using a fixed whole-plate configuration in which a camera mounted above the sample captured the full 35 mm plate at a constant camera-to-plate distance. Illumination was provided from below the plate stage, following the imaging approach described by Churgin and Fang-Yen [41] and Zhang et al. [17]. We have clarified these acquisition conditions in the Methods [Line 125-127], in addition to the camera model and resolution already provided (Table S1 and Figure S5), to make the imaging setup and its consistency across samples clearer.

#5: “The training procedure lacks detail: augmentation types, optimizer, learning rate schedule, batch size, and number of training epochs are not stated. Without these, the study is not reproducible.”

Response to #5: We have expanded the Methods section [Line 192-193; 201–205] for the initial training description, with all values taken directly from the saved training run artifacts. The revised Methods now specify that the models were trained for 50 epochs with a batch size of 1 and an input image size 1344 × 1344 pixels. Models were initialized from Microsoft COCO-pretrained weights distributed by the Ultralytics framework. We used Ultralytics ‘auto’ optimizer selection, which for our 231-image training set resolves to AdamW with initial learning rate of 0.01 and linear decay to a final learning rate of 0.0001. Momentum was set to 0.937, and weight decay to 0.0005, with a 3-epoch linear warmup.

#6: “YOLOv8 and YOLOv11 models are compared, but no statistical comparison (e.g., confidence intervals) is provided to determine whether observed improvements are meaningful and not within noise.”

Response to #6: We agree that point estimates alone are insufficient for a between-model comparison. We have computed 95% bootstrap confidence intervals for Precision, Recall, F1, and MAE of every model in Table 1 (1000 resamples of the 50-image held-out test set with replacement, with a fixed random seed so pairwise comparisons use identical resamples). We revised the Methods section to include a new statistical comparison paragraph, inserted after line 277 (end of the existing Statistical Analyses section), where we describe this procedure. The revised Table 1 reports each metric as a point estimate with its 95% CI. The 95% CIs of the top four models, YOLOv8-L, YOLOv9-E, YOLOv11-L, and the fine-tuned YOLOv11-L, overlap on all four metrics, and we inserted a new paragraph reporting this after the end of the Fine-tuning Results subsection [Line 350-358]. We explicitly note that statistical superiority over the top initial variants cannot be established with our 50-image test set, but we present the fine-tuned YOLOv11-L as our preferred pipeline on the basis of its consistent point-estimate ranking across Recall, F1, and MAE. This framing, in which model ranking is backed by explicit uncertainty estimates rather than point estimates alone, substantially improves the paper.

#7, #8, #9, #10: “The manuscript states that verified ground-truth counts exist but does not describe: How were annotations validated? Was inter-annotator agreement measured? This is critical when using biological imagery.”

Response to #7,8,9,10: Thank you for this important comment. We agree that the original manuscript did not clearly distinguish between annotation generation and annotation validation. We have revised the Methods to clarify that the ground-truth image set was annotated by a single annotator using manually drawn bounding boxes around each visible offspring, including overlapping individuals labeled separately. After annotation, the labeled images were manually reviewed for completeness and consistency, with particular attention to densely populated images and overlapping worms, before being used for model training and evaluation. Because all annotations were performed by a single annotator to maintain consistency in labeling criteria, inter-annotator agreement was not measured. We now state this explicitly in the revised manuscript [Line 166-169].

#11: “The manuscript shows counter-identity bias but does not clarify how many counters participated, nor whether counters were randomized or assigned systematically across blocks.”

Response to #11: Thank you for this comment. The original manuscript stated that manual counts were conducted by three trained human counters [Line 283], and we have added further information about how annotators were assigned. We have revised the Methods to clarify that each petri dish was manually counted once by a single counter and that dishes were assigned randomly among counters rather than systematically by experimental block [Line 284-286]. This additional clarification should make the basis of the counter-identity analysis clearer.

#12: “The claim that the YOLO model ‘outperformed manual counting in both speed and consistency’ is asserted but the manuscript does not quantify time saved (e.g., minutes per plate vs. seconds per inference).”

Response to #12: We have added a concrete quantitative comparison to the Discussion [Line 407-415]. The automated pipeline was benchmarked on the actual workstation (NVIDIA RTX 2080, CUDA 12.1, PyTorch 2.1, Ultralytics 8.3.64). Preprocessing, which includes Hough circle detection, grayscale conversion, 3 × 3 Gaussian blur, region-of-interest masking, and PNG save, as described in Methods lines 133–150, takes a mean of 731 ms per image (benchmarked on a random sample of 20 raw TIFFs after warmup). Fine-tuned YOLOv11-L inference takes a mean of 93 ms per image (batch size 1, image size 1344 × 1344; benchmarked on the 50 held-out test images after warmup). For the full 9,972-image dataset this corresponds to approximately 2 hours of preprocessing and 16 minutes of inference, for a total of approximately 2.3 hours of end-to-end automated processing. In comparison, manual counting of the same dataset was distributed across three trained human counters over approximately six months at approximately 10 minutes per image (including counting, examination of data, discussion, recounting).

#13: “The manuscript asserts that the pipeline is generalizable to other organisms, yet no evidence or demonstration is provided beyond C. elegans — this should be presented as a hypothesis, not a conclusion.”

Response to #13: agree that the original wording was too strong given that the present study evaluated the pipeline only using C. elegans images. We have revised the manuscript to present broader applicability as a hypothesis rather than as a demonstrated conclusion [Line 424-427]. Our goal here was to show that a YOLO-based object detection workflow can perform well for small-animal life-history assessment in a non-microscopic imaging setting. Because the workflow relies on general steps (image acquisition, manual annotation, and model training) rather than C. elegans-specific assumptions, we expect that the same general approach could be adapted to other systems after appropriate retraining and validation. Potential examples include related nematodes and other small-bodied organisms with similar imaging and counting challenges. We therefore revised the manuscript to clarify that any extension beyond C. elegans, including to other organisms or imaging modalities, remains a hypothesis for future work rather than a conclusion supported by the present data.

#14, #15: “The limitations paragraph mentions future extensions but does not discuss failure cases, such as: overlapping worms, debris mis-detection, variation in plate lighting.”

Response to #14, #15: Thank you for pointing this out. We have revised the limitations paragraph in Challenges and Future Opportunities section [Line 461-469] to mention likely failure cases, including overlapping worms, debris mis-detection, and variation in plate lighting or image quality. We also added clarification that the largest count discrepancies in our image set were concentrated in a small subset of images with mixed generations of nematodes or poor photo quality. Because these images were also challenging for manual counting, we note that some residual error likely reflects ambiguity in the source material rather than model-specific failure alone.

#16: “I think, if possible, The study would be significantly stronger with external-lab or field validation to demonstrate robustness across imaging and handling conditions.”

Response to #16: We agree that validation in an external laboratory or field setting would further strengthen the study by testing robustness across a broader range of imaging and handling conditions. The present work was designed to establish and evaluate the computer vision pipeline, so external validation was beyond the scope of this study. That said, we view such validation as an important next step. Other groups have expressed interest in using our approach, and our method was recently cited (e.g., Padilha, Matheus, et al. 2025). We are encouraged by this interest and eager to see the approach evaluated in additional systems and imaging environments in future work.

#17: “Consider defining abbreviations (YOLO, CV, precision/recall) at first use in both Abstract and Main Text.”

Response to #17: We have added the expansion of YOLO to its first occurrence in the Abstract [Line 29]. YOLO was already defined at first use in the Main Text [Introduction, line 74]. Precision and recall are defined in plain language at their first use in the Abstract (‘was correct about X% of the offspring it marked (precision)’ and ‘correctly detected Y% of all offspring visible (recall)’) and formally with equations in the Methods [Lines 237–242]. Mean absolute error (MAE) is defined at first use in the Methods [Line 221, line 247-249]. The abbreviation CV is not used in the Abstract or main text body; it appears only within the figure captions for Figures 3 and 5, where ‘computer vision (CV)’ is defined at first use within each caption.

#18: “Methods section would benefit from a workflow diagram summarizing complete pipeline.”

Response to #18: We agree

Attachment Submitted filename: Response_to_Reviewers.docx

# Peer review: Decision Letter 1

**DOI:** 10.1371/journal.pone.0354821.r003

**Authors:** Christian Braendle

22 Jun 2026

PONE-D-25-54218R1

From manual counting to YOLO: Using computer vision to automate large-scale fecundity assays in C. elegans

PLOS One

Dear Dr. Peng,

Thank you for submitting your manuscript to PLOS ONE. After careful consideration, we feel that it has merit but does not fully meet PLOS ONE’s publication criteria as it currently stands. Therefore, we invite you to submit a revised version of the manuscript that addresses the points raised during the review process.

We are pleased to inform you that your manuscript will be accepted for publication pending minor revisions. Therefore, we invite you to submit a revised version of the manuscript that addresses  three minor but useful points raised by reviewer 3:

Clarify the developmental stages for which the method has been validated, and discuss its potential limitations across different life stages.

Discuss the method’s performance at higher worm densities, including any associated limitations.

Address minor issues related to clarity and reporting, including the addition of missing units where appropriate.

Please submit your revised manuscript by Aug 06 2026 11:59PM. If you will need more time than this to complete your revisions, please reply to this message or contact the journal office at plosone@plos.org. When you're ready to submit your revision, log on to https://www.editorialmanager.com/pone/ and select the 'Submissions Needing Revision' folder to locate your manuscript file.

Please include the following items when submitting your revised manuscript:

- A letter that responds to each point raised by the academic editor and reviewer(s). You should upload this letter as a separate file labeled 'Response to Reviewers'.
- A marked-up copy of your manuscript that highlights changes made to the original version. You should upload this as a separate file labeled 'Revised Manuscript with Track Changes'.
- An unmarked version of your revised paper without tracked changes. You should upload this as a separate file labeled 'Manuscript'.

If you would like to make changes to your financial disclosure, please include your updated statement in your cover letter. Guidelines for resubmitting your figure files are available below the reviewer comments at the end of this letter.

If applicable, we recommend that you deposit your laboratory protocols in protocols.io to enhance the reproducibility of your results. Protocols.io assigns your protocol its own identifier (DOI) so that it can be cited independently in the future. For instructions see: https://journals.plos.org/plosone/s/submission-guidelines#loc-laboratory-protocols. Additionally, PLOS ONE offers an option for publishing peer-reviewed Lab Protocol articles, which describe protocols hosted on protocols.io. Read more information on sharing protocols at https://plos.org/protocols?utm_medium=editorial-email&utm_source=authorletters&utm_campaign=protocols.

As the corresponding author, your ORCID iD is verified in the submission system and will appear in the published article. PLOS supports the use of ORCID, and we encourage all coauthors to register for an ORCID iD and use it as well. Please encourage your coauthors to verify their ORCID iD within the submission system before final acceptance, as unverified ORCID iDs will not appear in the published article. *Only*  the individual author can complete the verification step; PLOS staff *cannot*  verify ORCID iDs on behalf of authors.

We look forward to receiving your revised manuscript.

Kind regards,

Christian Braendle

Academic Editor

PLOS One

Journal Requirements:

If the reviewer comments include a recommendation to cite specific previously published works, please review and evaluate these publications to determine whether they are relevant and should be cited. There is no requirement to cite these works unless the editor has indicated otherwise.

Please review your reference list to ensure that it is complete and correct. If you have cited papers that have been retracted, please include the rationale for doing so in the manuscript text, or remove these references and replace them with relevant current references. Any changes to the reference list should be mentioned in the rebuttal letter that accompanies your revised manuscript. If you need to cite a retracted article, indicate the article’s retracted status in the References list and also include a citation and full reference for the retraction notice.

[Note: HTML markup is below. Please do not edit.]

Reviewers' comments:

Reviewer's Responses to Questions

**Comments to the Author**

1. If the authors have adequately addressed your comments raised in a previous round of review and you feel that this manuscript is now acceptable for publication, you may indicate that here to bypass the “Comments to the Author” section, enter your conflict of interest statement in the “Confidential to Editor” section, and submit your "Accept" recommendation.

Reviewer #1: All comments have been addressed

Reviewer #2: All comments have been addressed

Reviewer #3: All comments have been addressed

**********

2. Is the manuscript technically sound, and do the data support the conclusions?

The manuscript must describe a technically sound piece of scientific research with data that supports the conclusions. Experiments must have been conducted rigorously, with appropriate controls, replication, and sample sizes. The conclusions must be drawn appropriately based on the data presented.

Reviewer #1: Yes

Reviewer #2: Yes

Reviewer #3: Yes

**********

3. Has the statistical analysis been performed appropriately and rigorously?

Reviewer #1: Yes

Reviewer #2: Yes

Reviewer #3: Yes

**********

4. Have the authors made all data underlying the findings in their manuscript fully available?

The PLOS Data policy requires authors to make all data underlying the findings described in their manuscript fully available without restriction, with rare exception (please refer to the Data Availability Statement in the manuscript PDF file). The data should be provided as part of the manuscript or its supporting information, or deposited to a public repository. For example, in addition to summary statistics, the data points behind means, medians and variance measures should be available. If there are restrictions on publicly sharing data—e.g. participant privacy or use of data from a third party—those must be specified.

Reviewer #1: Yes

Reviewer #2: Yes

Reviewer #3: Yes

**********

5. Is the manuscript presented in an intelligible fashion and written in standard English?

PLOS ONE does not copyedit accepted manuscripts, so the language in submitted articles must be clear, correct, and unambiguous. Any typographical or grammatical errors should be corrected at revision, so please note any specific errors here.

Reviewer #1: Yes

Reviewer #2: Yes

Reviewer #3: Yes

**********

6. Review Comments to the Author

Please use the space provided to explain your answers to the questions above. You may also include additional comments for the author, including concerns about dual publication, research ethics, or publication ethics. (Please upload your review as an attachment if it exceeds 20,000 characters)

Reviewer #1: All comments are addressed for this paper: 'From manual counting to YOLO: Using computer vision to automate large-scale fecundity assays.' Best of luck.

Reviewer #2: The manuscript presents its findings in a clear and organized manner and addresses a relevant research question. The objectives, methodology, results, and conclusions are generally well described and appear to be supported by the data presented. The study contributes useful information to the existing body of knowledge and may be of interest to researchers in the field. Based on my evaluation, I have no major concerns regarding the scientific content, research ethics, publication ethics, plagiarism, or dual publication. I have no additional comments or recommendations at this time and leave the final decision regarding publication to the editor's discretion.

Reviewer #3: Peng et al describe their approach to large-scale worm counting which uses a home-made imaging rig and the You Only Look Once (YOLO) automated object detection. They devote most of the manuscript to describing and quantifying the optimization of version and settings in the YOLO program, and comparing the accuracy of computer vision to that of hand-counting, which is currently the standard in the field. Their quantification of counter effect and experimental block effect alone is very convincing support for using computer vision to count worms. The authors' results are clearly explained, and publication of their optimizations will be a benefit to anyone who would like to automate worm counting in their own research.

I have one comment that I believe is important to address to ensure that this resource is useful to others (#1), and a couple additional minor comments/questions that I think would improve the quality of the manuscript, but would not be necessary for publication (#2,#3).

1. I don't believe the authors ever address limitations on what life stages can be imaged using this method. I assume that they are waiting until a set of eggs laid over 24 hours then develops into adults, because adults are easiest to image, but I wonder if earlier or mixed stages would be quantifiable as well. I'd be interested to see at what stage the reproducibility starts dropping off, even anecdotally, but at the very minimum I think it is important to mention that this was all done in adults (if that is true), so that people understand the limits of what's been tested.

2. Is there an upper limit for how many worms can be quantified on a plate? It seems that the images used in this manuscript cap out at about 200 worms, but I wonder if the authors can provide any guidance (again, even just anecdotally) on when counts become unreliable because of overlapping worms -- either due to large population sizes or clustering phenotypes seen in many wild isolates.

3. There are many values listed on lines 195-198 that have numbers but no units. Where possible, it may be useful to the reader to include units.

**********

7. PLOS authors have the option to publish the peer review history of their article (what does this mean?). If published, this will include your full peer review and any attached files.

If you choose “no”, your identity will remain anonymous but your review may still be made public.

**Do you want your identity to be public for this peer review?**  For information about this choice, including consent withdrawal, please see our Privacy Policy.

Reviewer #1: No

Reviewer #2: No

Reviewer #3: No

**********

[NOTE: If reviewer comments were submitted as an attachment file, they will be attached to this email and accessible via the submission site. Please log into your account, locate the manuscript record, and check for the action link "View Attachments". If this link does not appear, there are no attachment files.]

To ensure your figures meet our technical requirements, please review our figure guidelines: https://journals.plos.org/plosone/s/figures

You may also use PLOS’s free figure tool, NAAS, to help you prepare publication quality figures: https://journals.plos.org/plosone/s/figures#loc-tools-for-figure-preparation.

NAAS will assess whether your figures meet our technical requirements by comparing each figure against our figure specifications.

# Author comment: Author response to Decision Letter 2

**DOI:** 10.1371/journal.pone.0354821.r004

10 Jul 2026

We thank the editor and reviewers for their positive evaluation of our revised manuscript. We have addressed the three remaining minor points raised by Reviewer #3 by clarifying the developmental stages represented in the imaging dataset, adding discussion of limitations at higher worm densities and in strongly clustered populations, and clarifying reporting/units where appropriate. We also added Reference #42 and updated the funding statement as described in the cover letter. A detailed point-by-point response is included in the uploaded “Response to Reviewers” file.

Attachment Submitted filename: 2ndresponse_to_reviewers.docx

# Editor report: Decision Letter 2

**DOI:** 10.1371/journal.pone.0354821.r005

**Authors:** Christian Braendle

13 Jul 2026

From manual counting to YOLO: Using computer vision to automate large-scale fecundity assays in C. elegans

PONE-D-25-54218R2

Dear Dr. Peng,

We’re pleased to inform you that your manuscript has been judged scientifically suitable for publication and will be formally accepted for publication once it meets all outstanding technical requirements.

Within one week, you’ll receive an e-mail detailing the required amendments. When these have been addressed, you’ll receive a formal acceptance letter and your manuscript will be scheduled for publication.

An invoice will be generated when your article is formally accepted. Please note, if your institution has a publishing partnership with PLOS and your article meets the relevant criteria, all or part of your publication costs will be covered. Please make sure your user information is up-to-date by logging into Editorial Manager at Editorial Manager® and clicking the ‘Update My Information' link at the top of the page. For questions related to billing, please contact billing support.

If your institution or institutions have a press office, please notify them about your upcoming paper to help maximize its impact. If they’ll be preparing press materials, please inform our press team as soon as possible -- no later than 48 hours after receiving the formal acceptance. Your manuscript will remain under strict press embargo until 2 pm Eastern Time on the date of publication. For more information, please contact onepress@plos.org.

Kind regards,

Christian Braendle

Academic Editor

PLOS One

Additional Editor Comments (optional):

Reviewers' comments:

# Editor report: Acceptance letter

**DOI:** 10.1371/journal.pone.0354821.r006

**Authors:** Christian Braendle

PONE-D-25-54218R2

PLOS One

Dear Dr. Peng,

I'm pleased to inform you that your manuscript has been deemed suitable for publication in PLOS One. Congratulations! Your manuscript is now being handed over to our production team.

At this stage, our production department will prepare your paper for publication. This includes ensuring the following:

* All references, tables, and figures are properly cited

* All relevant supporting information is included in the manuscript submission,

* There are no issues that prevent the paper from being properly typeset

You will receive further instructions from the production team, including instructions on how to review your proof when it is ready. Please keep in mind that we are working through a large volume of accepted articles, so please give us a few days to review your paper and let you know the next and final steps.

Lastly, if your institution or institutions have a press office, please let them know about your upcoming paper now to help maximize its impact. If they'll be preparing press materials, please inform our press team within the next 48 hours. Your manuscript will remain under strict press embargo until 2 pm Eastern Time on the date of publication. For more information, please contact onepress@plos.org.

You will receive an invoice from PLOS for your publication fee after your manuscript has reached the completed accept phase. If you receive an email requesting payment before acceptance or for any other service, this may be a phishing scheme. Learn how to identify phishing emails and protect your accounts at https://explore.plos.org/phishing.

If we can help with anything else, please email us at customercare@plos.org.

Thank you for submitting your work to PLOS ONE and supporting open access.

Kind regards,

PLOS ONE Editorial Office Staff

on behalf of

Dr. Christian Braendle

Academic Editor

PLOS One
